# Another Lesson from Plants: The Forward Osmosis-Based Actuator

**DOI:** 10.1371/journal.pone.0102461

**Published:** 2014-07-14

**Authors:** Edoardo Sinibaldi, Alfredo Argiolas, Gian Luigi Puleo, Barbara Mazzolai

**Affiliations:** 1 Center for Micro-BioRobotics@SSSA, Istituto Italiano di Tecnologia, Pontedera, Italy; 2 The BioRobotics Institute, Scuola Superiore Sant'Anna, Pontedera, Italy; UMass, United States of America

## Abstract

Osmotic actuation is a ubiquitous plant-inspired actuation strategy that has a very low power consumption but is capable of generating effective movements in a wide variety of environmental conditions. In light of these features, we aimed to develop a novel, low-power-consumption actuator that is capable of generating suitable forces during a characteristic actuation time on the order of a few minutes. Based on the analysis of plant movements and on osmotic actuation modeling, we designed and fabricated a forward osmosis-based actuator with a typical size of 10 mm and a characteristic time of 2–5 minutes. To the best of our knowledge, this is the fastest osmotic actuator developed so far. Moreover, the achieved timescale can be compared to that of a typical plant cell, thanks to the integrated strategy that we pursued by concurrently addressing and solving design and material issues, as paradigmatically explained by the bioinspired approach. Our osmotic actuator produces forces above 20 N, while containing the power consumption (on the order of 1 mW). Furthermore, based on the agreement between model predictions and experimental observations, we also discuss the actuator performance (including power consumption, maximum force, energy density and thermodynamic efficiency) in relation to existing actuation technologies. In light of the achievements of the present study, the proposed osmotic actuator holds potential for effective exploitation in bioinspired robotics systems.

## Introduction

The question of how plants move in the absence of muscles has attracted the interest of many scientists in both past and current times [1]–[6]. From a biological perspective, the physiology of plant movement is central to the understanding of plant development and plant movement in response to environmental stimuli such as light and gravity [7]. Moreover, an understanding of these non-muscular movements could hold potential for developments in applied sciences and engineering, especially for the creation of novel biomimetic actuation strategies that are characterized by high energy efficiency and low power consumption [8]–[10].

From a physical viewpoint, plants can be considered highly complex hydraulic systems with a high level of compartmentalization, even at the microscopic level [11]. Plants are able to modulate their internal pressure in a selective way, by virtue of a finely regulated turgor-based strategy. Indeed, as a sort of “natural hardness”, turgor pressure 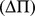 generated by water flux is sustained by an osmotic pressure difference: 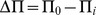, where 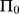 and 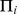 respectively represent the osmotic pressures (also called osmotic potentials) outside and inside the cell [12].

Osmosis is the chemo-physical phenomenon based on solvent transport involved in this mechanism and is normally described by equations strongly related to the chemical nature of osmolytes, i.e., the solute responsible for osmotic pressure differences. At a first approximation, the osmotic potential 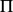 can be modeled through the classical Van't Hoff equation [13]:
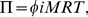
(1)where 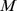 is the molarity of the osmolytes in solution, 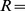
8.314 J K^−1^·mol^−1^ (the universal gas constant), 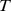 is the absolute temperature, and 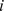 denotes the Van't Hoff coefficient, depending on the degree of osmolyte dissociation. Finally, 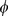 represents an empirical factor (often close to 1), which is introduced to enhance the accuracy of Eq. 1 by considering the non-ideal behavior of osmolytes [13]. Furthermore, solvent flux across a semi-permeable osmotic membrane is given by:
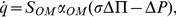
(2)where 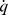 is the flux across the membrane, 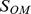 is the osmotic membrane surface, 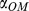 is the osmotic membrane permeability and 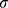 is an osmolyte rejection coefficient (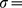
1 for an ideal membrane). 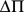 and 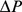 respectively denote the osmotic pressure difference and the pressure difference between the two sides of the osmotic membrane. According to Eq. 2, it is possible to define forward osmosis when 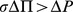 and reverse osmosis when 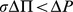 [14]. In addition, 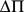 can be generated by several physical mechanisms, including direct control over the osmolyte concentrations (an example given by solute and solvent injection), electric fields (electro-osmosis), and chemical reactions able to modify osmolytes concentrations.

In plants, osmosis acts through the coordinated action of a multitude of simple cell-level units, relying on four main elements: an osmotic membrane, a compliant structure also acting as transducer, a rigid structure, and an available osmolyte able to build up the osmotic power reservoir and to be efficiently transported to all plant tissue regions. Indeed, water distribution “osmotically pumped” by osmolytes in the microscopic compartments of plant tissue is responsible for the turgor distribution along the plant organism. At the plant cell-level, this turgor is built owing to the stiff plant cell wall, which is made of highly organized cellulose microfibrils embedded in a pectin matrix [15]. Plants transform the isotropic stress produced by turgor pressure into directional movements at the cellular, tissue and organ level, thanks to the inhomogeneity and anisotropy of the cell wall, tissue and organ textures [4], [15].This is achieved by coupling the compliant compartments involved in the osmotic process with suitably rigid structures, such as lignin-rich and dead tissues organized into metastable spring-like systems, or by directing the osmotic flux through a strict biochemical mechanism involving active osmotic enhancers and suppressors (e.g., the auxin mechanism, special osmotic metabolism, and osmotic agent active transport) [4]. Furthermore, it is worth noting that plants evolved to exploit an impressive variety of osmolytes: inorganic electrolytes (mainly potassium nitrate, sodium chloride, and sodium hydrogen phosphates) from the soil [16], carbohydrates (glucose, fructose, sucrose and other trace present carbohydrates) obtained by photosynthetic internal processes, and amino acids and peptides present in the cytosol environment [17]. Finally, the osmolyte concentrations can be controlled by an electro-osmotic mechanism triggered by an action potential [18] or environmental changes (e.g., moisture, light and metabolic modifications) that affect the osmotic potential, possibly in a purely passive way [19].

Plant movements can be classified into several categories, such as nastic (movements independent of the spatial direction of a stimulus) or tropic (movements influenced by the direction of a stimulus), active (movements produced by live plants that activate and control their responses by moving ions and by changing the permeability of membranes based on potential actions) or passive (movements mainly based on dead tissue that can undergo predetermined modifications when the environmental conditions change), and reversible (movements specifically based on reversible variations in the turgor pressure) or irreversible (movements that can happen only once) [20]. An additional classification has been recently proposed [21], which exploits the timescale for water transport through plant tissue in order to distinguish between purely water-driven movements (growth, swelling/shrinking) and those that use mechanical instabilities to amplify the capacity to move.

The movements associated with mechanical instabilities are faster than the water-driven ones, and a suitable boundary between the two categories is provided by the fastest timescale for swelling. However, a separate treatment is needed for unicellular movements (water flow at the cellular level) and multicellular movements (water flow at the tissue level), since the permeability associated with water transport slightly varies depending on the lengthscale [4]. On the one hand, the fastest cell swelling is associated with the so-called cell relaxation time 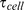, defined as follows [4]:
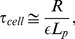
(3)where 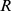 denotes the mean cell radius (obtained from the volume-to-surface ratio), 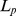 is the cell membrane conductivity, and 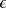 denotes the bulk modulus of the cell, which reflects both the elastic properties of the cell wall and the geometry of the cell. On the other hand, the fastest tissue swelling is associated with the so-called poroelastic time 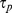, defined as follows [4], [21]:
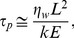
(4)where 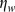 denotes water viscosity, 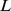 indicates a typical tissue size (defined as the smallest macroscopic moving part), 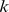 is the Darcy permeability of the porous tissue, and 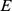 denotes the elastic Young modulus of the tissue. These two timescales set the fastest possible water-driven processes in plant cells and tissues and thus can be used as guide for designing osmosis-based actuators in a biomimetic approach.

**Figure 1:**
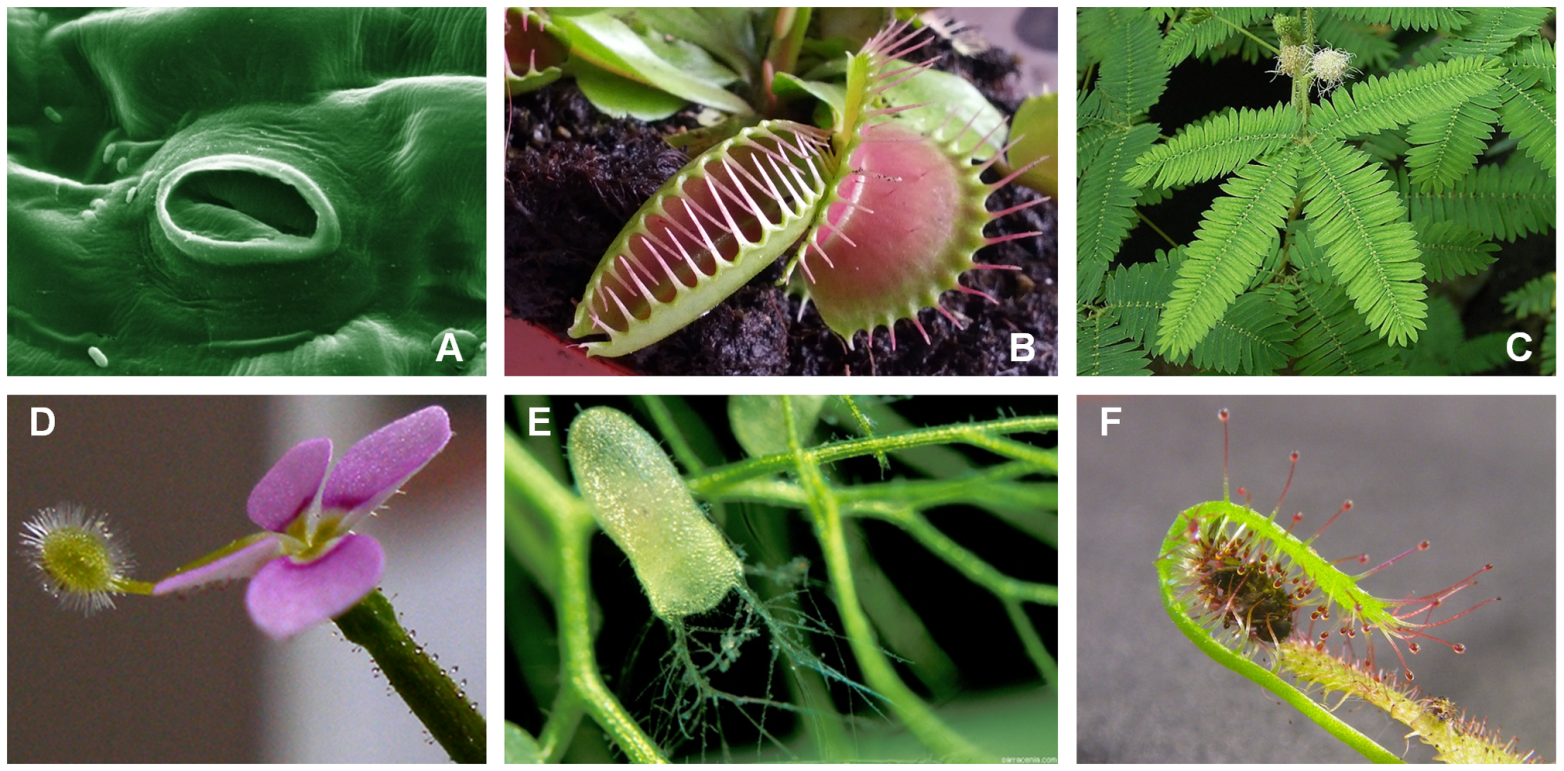
Examples of osmosis-based movements in plants. (A) Stomata guard cells. (B) *Dionaea muscipula* leaf traps. (C) *Mimosa pudica* leaves. (D) *Stylidium debile* flower, with protruded column. (E) *Utricularia inflata* door traps. (F) *Drosera capensis* tentacles (with caught prey). Credits: (A,D) www.wikipedia.org; (C) www.wordpress.com; (E) http://calphotos.berkeley.edu; © Barry Rice 2006.

In this work, we considered active and reversible osmosis-based movements as source of inspiration. Amongst the unicellular examples, the most famous is provided by the opening and closure of the stomata, which are small pores on the surface of the leaves that control the leaf transpiration and gas exchange with the atmosphere. In most plants, the stomatal complex consists of two kidney-shaped guard cells that flank a central pore (Figure 1A). The pore opens when the turgor pressure inside the guard cells increases (up to nearly 5 MPa) owing to accumulation of solute, while it closes when turgor decreases, since the two guard cells are pressed together by the surrounding cells. The stomatal movement is purely water-driven, and the bending of the guard cells originates from the large asymmetry in wall thickness and the mechanical anisotropy of the cellulosic network [4]. Amongst the multicellular rapid and active movements, let us mention the following examples: *Dionaea muscipula* (Venus Flytrap), *Utricularia* (bladderwort), *Stylidium debile*, and *Mimosa pudica* (Figure 1B–E). In all of them, movements are activated by touch stimuli (thigmotropism), with the exception of *Mimosa pudica*, which also closes its leaves in response to light (phototropism) and heat stimuli (thermotropism). Moreover, a stimulus-motion phase (e.g., leaf/trap closure) is systematically followed by a reset phase required to recharge the actuation machinery. The stimulus-motion phase is associated with mechanical instability [22], even if the mechanisms at the microscopic level by which the plant actively overcomes the instability threshold are still debated [6]. Conversely, the reset phase is water-driven and occurs over longer timescales, namely hours to days for *Dionaea muscipula* [19], 20–60 min for *Utricularia* [23], [24], nearly 10 min for *Stylidium debile* and *Mimosa pudica* [19]. Moreover, it takes hours to also reset the movement of *Drosera* [19], a carnivorous plant that wraps its prey for killing and digestion through glandular tentacles (Figure 1F).

We further focused on unicellular movements, by addressing a single compartment actuator that mimics the water-driven movement of a giant plant cell, both for simplicity and because it can provide a fundamental unit for more complex osmosis-based actuation systems. The main aim of this study was to develop a low-power-consumption, forward osmosis-based actuator that is capable of generating suitable forces during a characteristic time on the order of a few minutes. Such an actuator does not currently exist: the only prior art is represented by a miniature osmosis-driven, irreversible piston mechanism that combines drug release and mechanical actions (less than 10 N) to produce bone distraction over nearly 200 h [25]. On the other hand, the achievement of the pursued actuator is very appealing for developing many bioinspired robotics systems, since the integration of power-cheap yet effective actuation strategies is an issue. However, due to the founding character of this study, we did not address actuator reversibility, in order to focus on relevant aspects of the osmotic process while tackling technical issues in an incremental manner.

For completeness, let us mention that the osmotic principle has been considered for actuating several artificial systems. For instance, electro-osmotic pumps have been developed for microfluidic [26] and lab-on-chip applications [27]. Moreover, electro-osmosis has been used for driving protein transporters (extracted from plant cell membranes) across the artificial membrane of micro-actuators [28], as well as for steering the tip of a mechatronic system inspired to the plant roots apices [10] for the purpose of soil exploration and monitoring. In contrast, forward osmosis has been used in fewer artificial systems that operate through direct osmolyte concentration control. Most of them have been developed in the biomedical context, particularly as miniature pumping systems able to achieve a constant drug release rate over a prolonged time period [29]. Furthermore, the use of chemical reactions to control osmolyte concentrations has also been described in a few conceptual works, especially for continuous flow systems in which the osmotic pressure differences are caused by biochemical [30] or catalytic reactions [31]. Finally, some additional examples of plant-inspired actuation devices have been recently developed [32]–[34], which exploit a mechanism very close to forward osmosis, namely the diffusion/evaporation of solvent through membranes or polymer networks.

Hereafter we report the design, fabrication and experimental assessment of the novel osmotic actuator based on plant-inspired forward osmosis. In more detail, we first present two actuator concepts, and we provide some estimates for their performance, including the timescale. Once selected the actuation concept involving bulging deformations, actuator design and fabrication are briefly discussed, together with the experimental protocols (free bulging tests and actuation force measurements) used for its assessment. Results are then reported, regarding in particular the achieved dynamics and force rates, also in relation to model predictions. Furthermore, we discuss the performance of the osmotic actuator, in particular by comparing its timescale with that one of a typical plant cell. Finally, we discuss other performance indicators (such as power consumption, maximum actuation force, energy density and thermodynamic efficiency), also in relation to existing technologies, in order to highlight the potential for effective exploitation of the proposed technology.

## Materials and Methods

**Figure 2:**
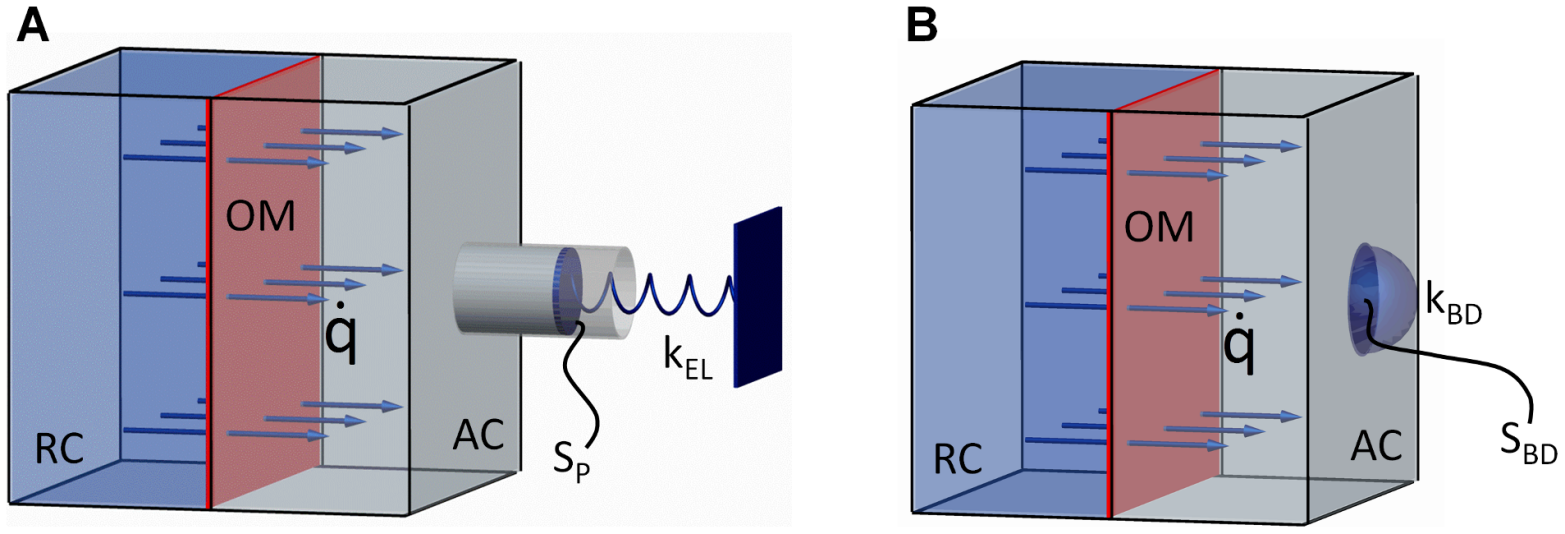
Osmotic actuator concepts. Solvent flows from the reservoir chamber (RC) to the actuation chamber (AC) through the osmotic membrane (OM), and actuation work is gathered/transduced either (A) through the elastic deformation of an external (piston-like) load, or (B) through the bulging deformation of a disk-shaped portion of the actuation chamber boundary. The developed actuator is based on the concept in (B), since it is more closely related to the plant cell model inspiring this study. (Additional symbols are discussed in the main text.)

Starting from an analysis of osmotic actuation in plants [35], our actuator consists of four main elements: a reservoir chamber (RC), an osmotic membrane (OM), and an actuation chamber (AC) featuring both a rigid boundary and a compliant boundary. The latter is also used to transduce the actuation force. Furthermore, the driving osmotic potential is provided by the initial concentration of osmolytes in the AC, which draw water (our working solvent, chosen for its simplicity and environmental compatibility) from the RC. In more detail, let us preliminarily introduce the two concepts sketched in Figure 2: the one in Figure 2B was directly used to design the actuator, while that one in Figure 2A was recalled when analyzing some of its performances. These concepts are thoroughly modeled in Files S1 and S2, where the corresponding osmotic actuation dynamics and performance are quantitatively analyzed. Nevertheless, key model results are concisely reported below, so as to highlight the model-based approach adopted for designing and characterizing the actuator, besides allowing for an almost self-contained presentation.

In Figure 2A the osmotic actuation work is gathered/transduced through the elastic deformation of an external load, namely a piston with surface area 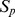 and stiffness 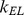, so that the pressure 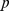 in the AC satisfies the relation 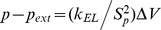, where 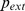 denotes the external pressure, and 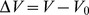 is the AC volume increment caused by the water flux (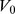 being the initial AC volume). Differently, in Figure 2B the osmotic actuation work is gathered/transduced through the bulging of an elastomeric disk with surface area 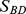 and stiffness 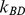, such that 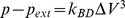. The latter relation, which is classically introduced when considering small bulging deformations of an elastic membrane [35], [36], can be also adopted for the bulging at hand, see File S2. Let us remark that we designed the actuator based on the bulging concept since it more closely derives from the plant cell model inspiring our study. In this regard, let us remark that 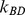 accounts for both the material (elastic) and geometrical properties (thickness and surface area) of the bulging disk, so that the actuator volumetric stiffness 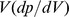 depends on both the elastic and the geometrical properties of the compliant AC boundary, together with the AC size. This aspect is fully consistent with the plant cell model. Indeed, the cell bulk modulus 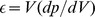, which was already introduced in Eq.3 and which characterizes how changes in cell volume are related to changes in turgor pressure, depends on both the elasticity of the cell wall and on the cell geometry [4].

Relevant performance indicators for the considered osmotic actuation concepts are detailed in Files S1 and S2, yet approximate expressions are reported in Table 1, for ease of presentation. They are more accurate for 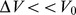, as occurring e.g. for stiff enough external loads or bulging disks. In more detail, we provide relevant estimates for actuation timescale, maximum force, initial force rate, peak power, power density, actuation work (i.e. energy stored through the displacement of the compliant AC boundary), energy density, and thermodynamic energy efficiency. As for the latter quantity, 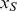 denotes the solute molar fraction; moreover, 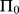 represents the osmotic potential associated with the initial solute concentration (all the other symbols were previously defined).

**Table 1:** Actuator performances, for both the work transduction concepts (elastic external load, disk bulging).

Performance indicator(a)	Elastic external load		Bulging	
Timescale [s]	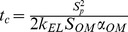	(5)	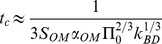	(13)
Maximum force [N]	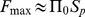	(6)	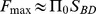	(14)
Initial force rate [N s^−1^]	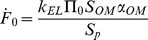	(7)	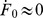	(15)
Peak power [W]	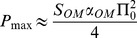	(8)	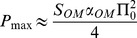	(16)
Power density [W m^−3^]	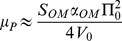	(9)	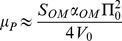	(17)
Work (stored energy) [J]	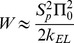	(10)	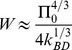	(18)
Energy density [J m^−3^]	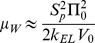	(11)	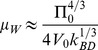	(19)
Thermodynamic energy efficiency [-]	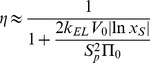	(12)	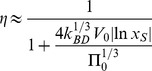	(20)

(a) The approximations are more accurate for small volume increments of the actuation chamber, as occurring e.g. for stiff enough external loads or bulging disks. (More general expressions are reported in Files S1 and S2.)

The timescale 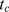 of the pursued osmotic actuator is provided by Eq.13. It should be noticed that such a timescale depends on the OM permeability, on the AC size (basically through the OM surface area), on the stiffness 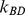 of the movable AC boundary (based on both elastic and geometrical properties of the bulging disk), and on the initial solute concentration. It is worth observing that the AC volume-to-surface ratio does not directly affect this timescale, differently from what happens for plant cells, because in our concept water only flows through the OM, which is assumed to behave like a rigid boundary, while the AC boundary displacement is only associated with the bulging disk. We therefore split the permeability and the compliance characteristics of the AC boundary, differently from the plant cell model where the cell wall can simultaneously serve to both functions. We started the actuator design by targeting a typical size around 10 mm, since it should permit to achieve characteristic actuation times on the order of a few minutes, according to the preliminary estimates in [35]. Then, based on some preliminary design choices, Eq.13 was used to assess whether the considered elastomeric disks were suitable to achieve the sought timescale. Actuator design and fabrication are concisely presented below (a detailed discussion is reported in File S3), together with experimental methods used to measure selected performances, namely the actuation timescale and the initial force rate.

### Actuator design and fabrication

Sodium chloride (NaCl) was preliminarily chosen as osmolyte, for the following reasons: it is perfectly dissociated in water; NaCl water solutions are indefinitely stable and permit osmotic pressure differences on the order of several MPa (effectively usable for many actuation tasks); manufacturing technology to produce osmotic membranes suitable for operation with NaCl is known, optimized and reproducible; NaCl is inexpensive, easily available, and produces no dangerous waste.

Moreover, AISI 316 stainless steel was chosen as main structural material, due to its enhanced corrosion resistance in chloride-containing environments, while Plexiglas was chosen for the RC part not in contact with the AC, to facilitate visual inspection. Moreover, a semi-permeable forward-osmosis membrane specifically designed for operation with NaCl was purchased from HTI (Hydration Technology Innovations, Scottsdale, AZ, USA), with water permeability 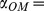
3·10^−13^ m s^−1^ Pa^−1^, rejection coefficient 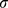 in the range 0.95–0.97 (thus close to the ideal case), and very low performance degradation due to NaCl fouling. We also fixed 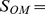
100 mm^2^, and a 5 mm diameter hole for the bulging disk protrusion (so that 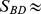
20 mm^2^). Then, we identified two commercially available elastomers, namely AT 31 F rubber (SIGAP, Brescia, Italy) and Viton (Camthorne Industrial Suppliers, Stoke on Trent, UK), for the bulging disk. These elastomers were selected out of a larger set of initial candidates, based on the following preliminary assessments. They showed suitable strength and minor inelastic behavior under standard stress-strain tests (Instron Model 4464, ITW Test and Measurement Italy S.r.l., Pianezza, Italy; tests repeated in triplicate; data recorded with Labview 8.6, National Instrument, Austin, TX, USA, and processed with Matlab, Mathworks, Natick, MA, USA). Moreover, once fixed a 1 mm thickness for the disk, samples with a 10 mm diameter (large enough to be glued to the AC body) showed no leakages during preliminary bulging tests. In addition, the stiffness coefficient 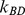 was evaluated through an indentation test (same equipment as above), in particular by measuring the force 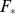 needed to produce a hemispherical bulge with volume 
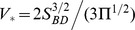
 (see Files S2 and S3 for details). Then, Eq.13 was used to estimate the corresponding actuation timescale 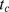, by assuming a 1 M NaCl solution (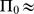
5 MPa). It turned out 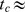
2 min for both elastomers, in agreement with the targeted timescale, so that we considered bulging disks made of both materials.

**Figure 3:**
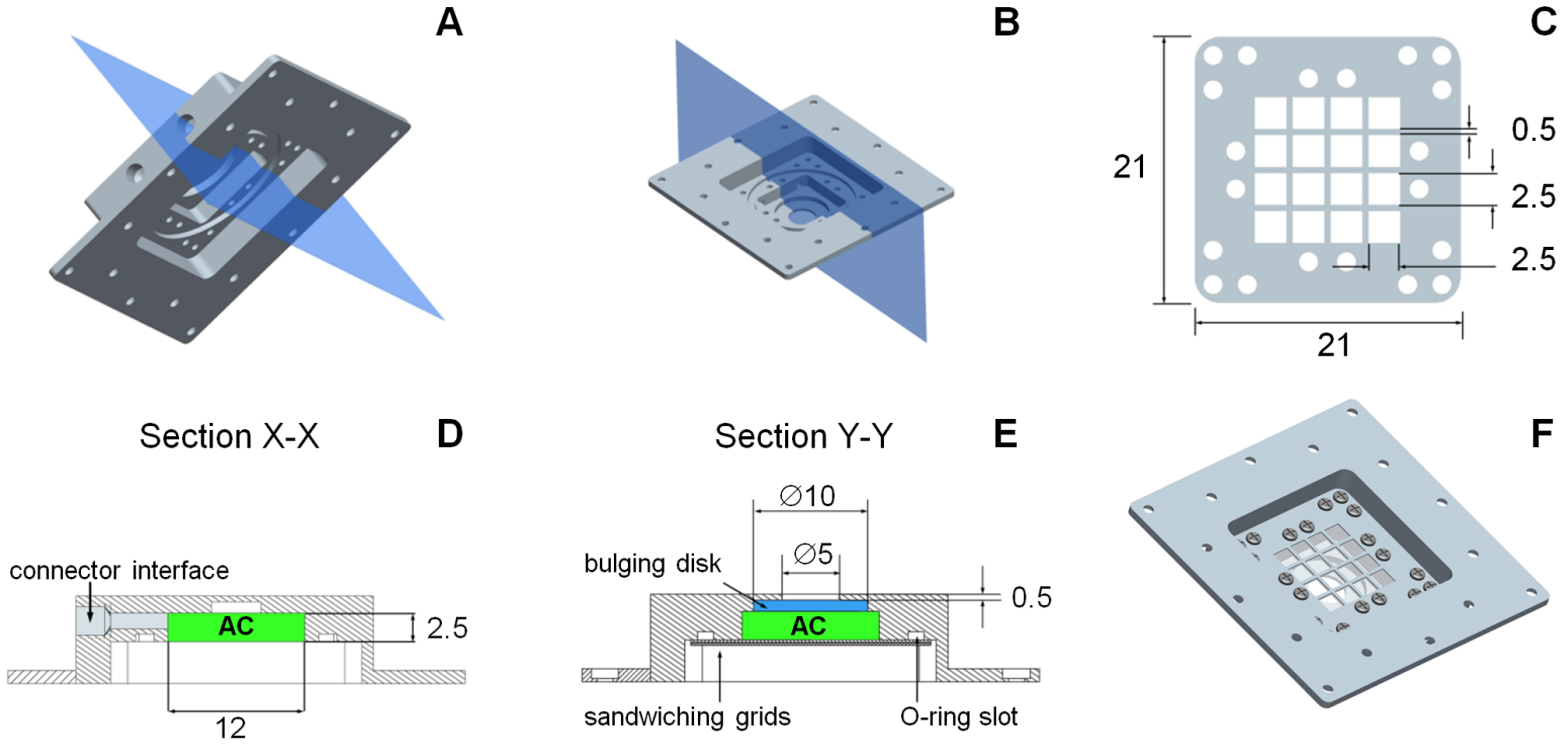
Design details of the actuation chamber module. (A,B) 3D views of the actuator module hosting, in particular, the actuation chamber. (C) Top view of an osmotic membrane sandwiching grid, also showing the passing-through holes for the fastening screws. (D) Section showing the interface for the loading/flushing connectors. (E,F) Section and 3D view of the main body of the module, also featuring the sandwiching grids. The bulging elastomeric disk is sketched in (E), for ease of illustration. Dimensions are shown in mm.

**Figure 4:**
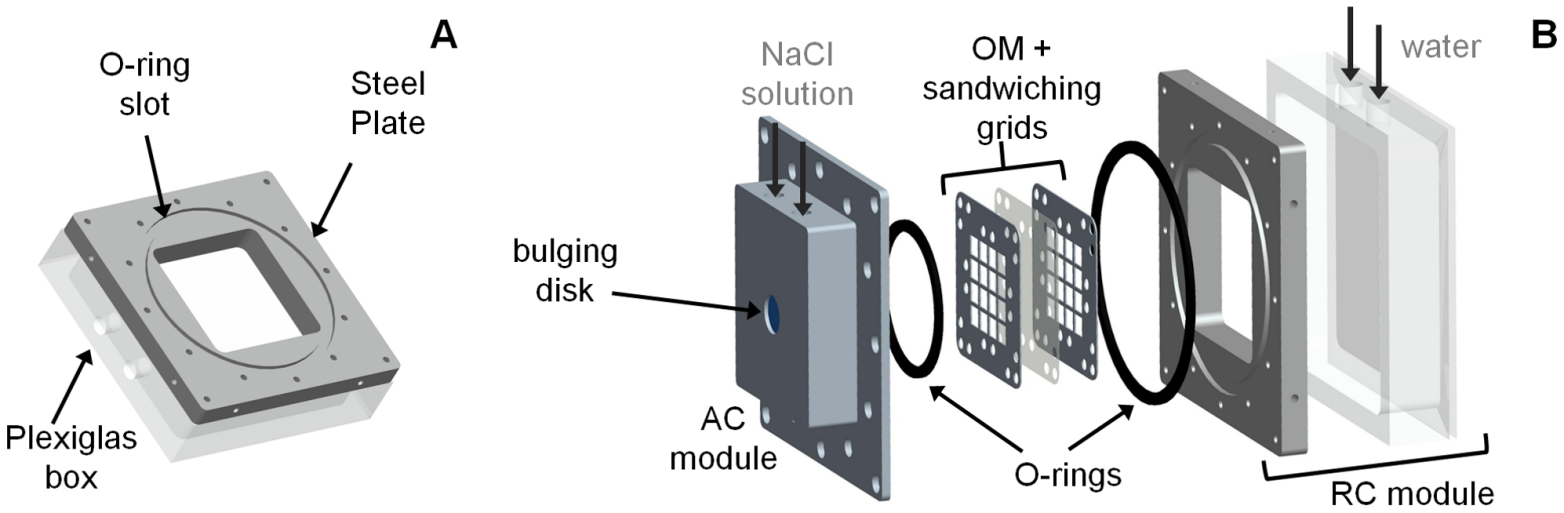
Design details of the reservoir chamber module and actuator assembly. (A) 3D view of the reservoir chamber module. (B) Exploded view of the main components of the actuator.

We then defined the actuator geometry, by designing two modules: one devoted to the RC, the other hosting the AC and including the OM and the elastomeric disk (that provided part of the AC boundary). The AC module is shown in Figure 3. It features, in particular, two supporting grids (Figure 3C,E) that sandwich the OM to contain its deflection (to prevent performance loss, see [35]); their size was defined also based on finite element analysis (Abaqus 6.11, Dassault Systèmes, Velizy-Villacoublay, France). A 12×12×2.5 mm working volume resulted for the AC. Channels for the osmotic solution loading/flushing were also designed (Figure 3A,D), based on commercially available fluidic connectors. Furthermore, a cylindrical slot (10 mm diameter, 1 mm height) was foreseen for the bulging elastomer (Figure 3E), to be glued to an annular surface (Figure 3B) adjacent to the cylindrical hole allowing for bulge protrusion. The wall thickness for the annular surface was 0.5 mm (Figure 3E), so that the bulge protrusions could be visually detected for bulge profiles higher than 0.5 mm. Potential leakages were prevented through an O-ring seal, whose slot is visible in Figure 3B,D,E. Sealing was achieved by fastening the OM sandwiching grids through precision screws (slots and passages shown in Figure 3A,B,C). A 3D view of the considered module also featuring the fastened grids is shown in Figure 3F; a flange is also shown, which was introduced for proper interfacing with the RC module, consisting of a steel plate and a Plexiglas box (Figure 4). The plate features an O-ring seal, and slots for the fastening screws used to seal (Figure 4A); additional screw holes were added to the lateral surface to connect the actuator to any external supports. The Plexiglas box features holes for water loading/flushing, and allows for visual inspection thanks to the slot in the steel plate (Figure 4A). An exploded view of the main components of the actuator is shown in Figure 4B.

As regards fabrication, laser cutting (CO_2_ VERSAlaser VL S2.30 system, Laser&Sign Technology, Sidney, Australia) was used for the RC Plexiglas components, subsequently glued to one another and to the steel plate through bi-component epoxy glue. The same laser-cutting machine was exploited for profiling the OM and for the elastomeric disk, which was subsequently glued to the main body of the AC module with polychlorobutadiene glue. Furthermore, YAG laser cutting (SL 800 HS, LPKF Laser & Electronics, Garbsen, Germany) was used for the OM sandwiching grids. Finally, the AISI 316 stainless steel parts were fabricated using a CNC 5 axis micro-milling machine (HSPC, KERN-Microtechnic, Eschenlohe, Germany).

### Actuator performance assessment

Let us preliminarily recall that, although we foresaw AC flushing in view of multiple actuations, in this study we did not address actuator reversibility, to contain the device complexity; hence, the performance of a single activation was deliberately investigated. The height of the bulging protrusion versus the time was measured during free bulging tests, by varying the initial solute concentration in the AC. The main aim of these tests was to measure the actuation characteristic time. Moreover, the force exerted by the actuator versus the time was recorded through a load cell, for several values of the initial solute concentration. These tests were not primarily meant to measure the maximum achievable force (because in a specific application it clearly depends on the actuated load as well), even if were interested in reference force values. They were rather aimed to measure the dependence of the initial force rate on the solute concentration, so as to gain further confidence in the actuator characterization, and thus on its potential performance.

The characteristic time of the bulging actuator was defined as the time interval needed to obtain a hemispherical bulge (since the characteristic length for bulge protrusion was implicitly set by the size of the corresponding hole). In light of the adopted sizes, the bulge was hemispherical when the protrusion height was equal to 2 mm. The OM was preliminarily prepared by removing its vegetable-based glycerin protective layer by soaking it in deionized water for 30 min. After glycerin removal, care was taken to prevent the OM from drying out or freezing. Then, the OM was sandwiched between the dedicated grids and fastened to the AC module, taking into account the flux directionality. The AC was then filled with an NaCl water solution with the desired molarity; such solutions were prepared in advance to achieve perfect solution conditioning, and then stored in the fridge to avoid biocontamination. AC loading was performed via manual syringe injection (for simplicity, the fluidic connectors were not used for such basic tests), and sealing was achieved through proper screws. Care was taken during the loading of the osmotic solution to prevent the formation of visible bubbles in the AC, whose compressibility could negatively affect the actuator performance. Moreover, sealing was carefully performed so as not to induce any preload. Once the actuator was placed on a dedicated support having a millimeter-scale grid on the background, distilled water was injected into the RC, and the resulting bulging was optically recorded (HDR-CX220E, SONY, Tokyo, Japan). Video frames were then extracted and processed through Matlab (Mathworks, Natick, MA, USA), and the bulge protrusion height was detected by manual image processing (image resolution over 28 pixel/mm). For each of the considered elastomers, 2 NaCl concentrations (1 M and 2 M) were considered, and all tests were carried out in triplicate. Video recording was stopped a few minutes after obtaining the sought hemispherical shape for the bulge. All tests were carried out at room temperature (25°C).

As regards force rate measurements, once prepared the actuator as described above, it was tightly and rigidly fixed to a dedicated support, which hosted a load cell with maximum capacity of 500 N and a maximum system error tolerance of ±1% (LLB250, Futek Advanced Sensor Technology Inc., Irvine, CA, USA). In particular, coupling was such that the bulging disk and the load cell sensor were initially put in contact, while avoiding any preloads. Force measurements were collected with the test and measurement software Sensit (Futek Advanced Sensor Technology Inc., Irvine, CA, USA) with 1 acquisition per second and processed with Matlab (Mathworks, Natick, MA, USA). For each of the considered elastomers, NaCl concentrations of 1 M and 2 M were considered, and all tests were carried out in triplicate. Despite being interested in the initial build-up of the actuator force, recordings were prolonged over 10 min. All tests were carried out at room temperature (25°C).

## Results

**Figure 5:**
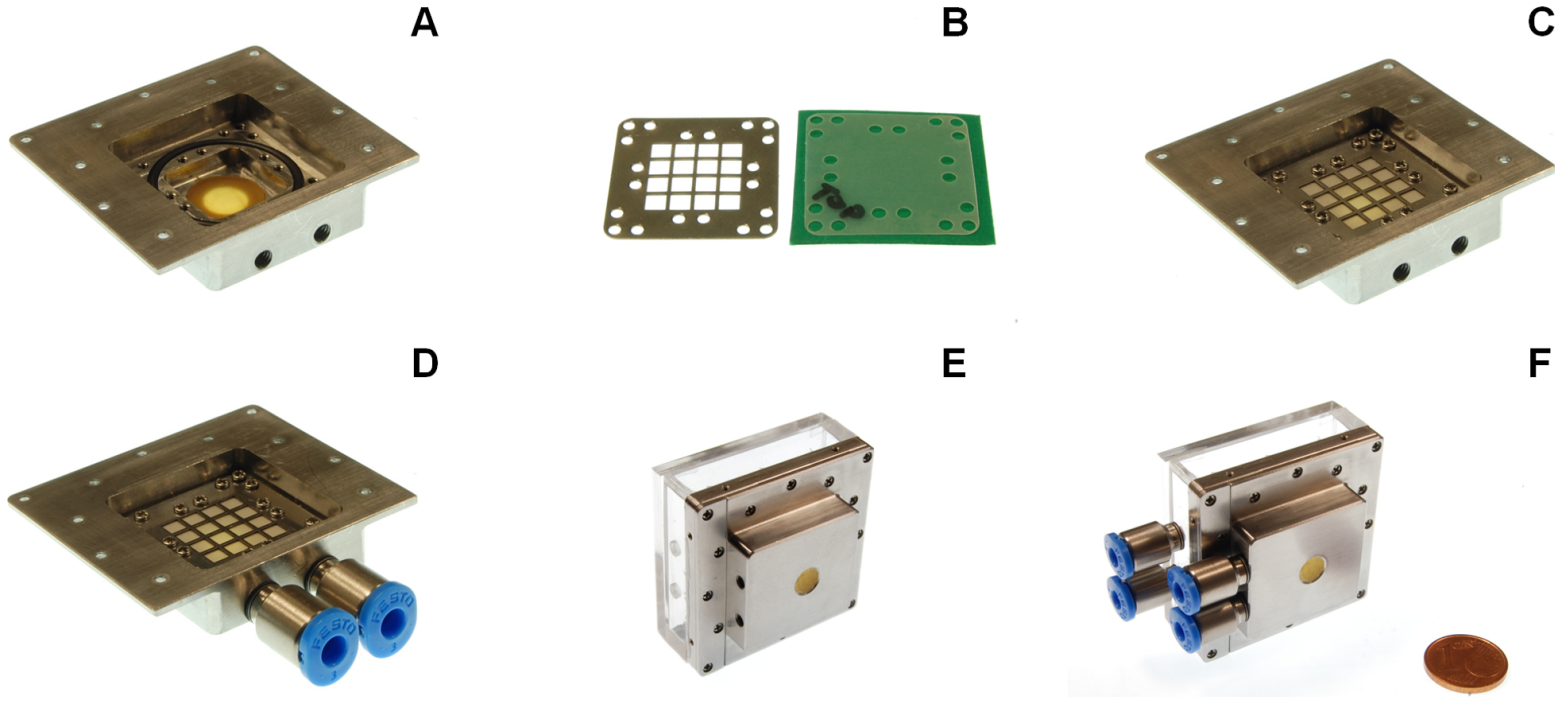
The assembly and components of the osmotic actuator. (A) Micro-machined body of the actuation chamber; the bulging elastomeric disk and the O-ring seal are also visible. (B) Laser cut osmotic membrane and sandwiching grid. (C) Assembly of the actuation chamber module, (D) also featuring the fluidic connectors. (E) Osmotic actuator assembly (F) also featuring the fluidic connectors.

Figure 5 shows the osmotic actuator. In more detail, Figure 5A shows the micro-milled body of the actuation chamber module, with a glued bulging disk. The laser-cut osmotic membrane is shown in Figure 5B, along with one of the two sandwiching grids, while the corresponding assembly is shown in Figure 5C, as fastened through the chosen micro-screw configuration. Figure 5D then shows the actuation chamber module assembly, also featuring the loading/flushing connectors. Finally, Figure 5E depicts the actuation chamber module (without connectors) and the reservoir chamber module fastened to each other, while the complete actuator is shown in Figure 5F.

**Figure 6:**
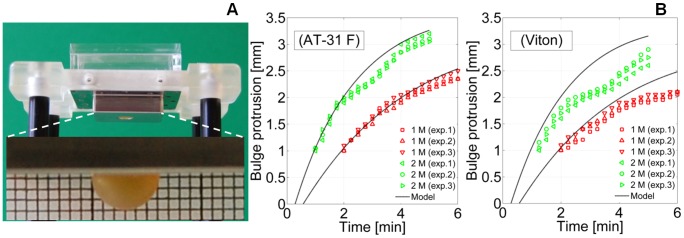
Free bulging tests results. (A) Part of the experimental set-up used for the bulging tests; (inset) exemplificative bulge (2 M NaCl solution, AT 31 F elastomeric disk). (B) Bulge protrusion height versus time. Experimental measures (in triplicate) and theoretical model predictions are both shown.

The results of the free bulging tests are shown in Figure 6, while an exemplificative bulging dynamics is shown in Video S1. In particular, Figure 6A depicts the details of the experimental set-up, while Figure 6B reports bulge protrusion versus the time. The hemispherical bulge shape (corresponding to a bulge protrusion height of 2 mm) was achieved in 4–5 min for the 1 M NaCl solutions, and 2–3 min for the 2 M solutions. We therefore succeeded in achieving the targeted actuation characteristic time. To the best of our knowledge, this is the fastest response of any forward osmosis-based actuator that has been reported in the literature to date [25], [29]. It should be noted that we observed similar trends for both elastomers, although the Viton elastomer exhibited a stiffening behavior after reaching the hemispherical bulge shape. This was not surprising because Viton is a silica perfluorinated polyisoprene composite whose mechanical behavior at higher strains can suffer from instabilities related to phase separation. This implied slightly longer characteristic times compared to those of the AT 31 F rubber, which achieved a hemispherical bulge in either 4 or 2 min depending on the initial molarity (yet this did not weaken the achieved result). The measured protrusion heights in Figure 6B start from 1 mm because optical effects occurred when the bulge first came out of the actuation chamber, also due to the reflection of light by steel. These effects worsened the quality of the corresponding images, thus rendering the image processing less straightforward.

Remarkably, we were able to also predict the bulge dynamics, using the theoretical model detailed in File S2 (from which Eq.13–20 originate in the case of small bulging deformations). The predicted trends are marked by solid lines in Figure 6B: they do not start from the axes origin because it takes time for the bulge to protrude out of the external surface of the actuator. They are in excellent agreement with the experimental results for the AT 31 F elastomer: its characteristic time was accurately predicted (approximately 4 min and 2 min for the 1 M and 2 M solutions, respectively), and its bulge height trend was also predicted (with a maximum discrepancy for the 1 M solution at 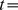
4 min and for the 2 M solutions at 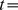
2 min, respectively equal to 0.10 mm and 0.13 mm). Very good agreement was also achieved for the Viton elastomeric disk because the observed stiffening only marginally affected the initial part of the deformation. Indeed, the model highlighted the fact that the first part of the bulge dynamics was mainly ruled by the initial solute concentration, so that 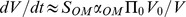. This explained the very similar initial trend for both elastomers, and the fact that the characteristic time roughly scales with the initial salt molarity. These results strengthened our confidence in the model-based approach used for designing the actuator.

**Figure 7:**
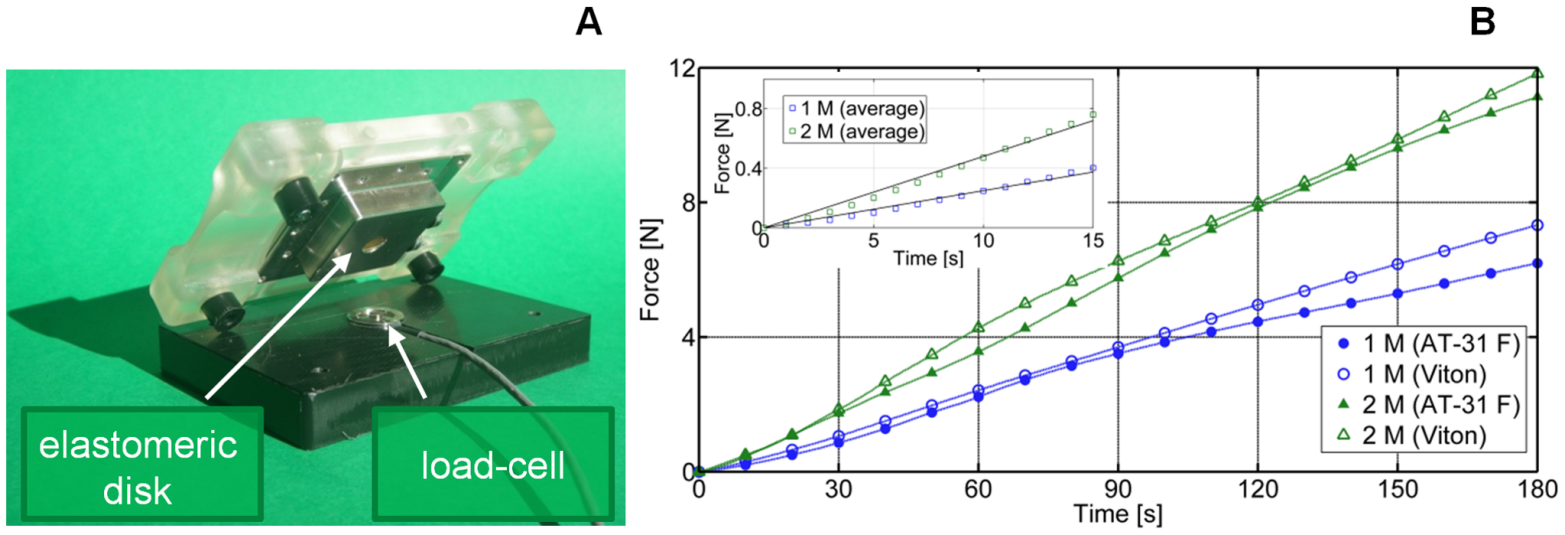
Force measurement results. (A) Set-up (unfastened) used for the osmotic actuator force measurements. (B) Measured forces (from triplicate experiments) for both the elastomeric disks and the 2 solute concentrations. The average trend for each solute concentration is shown in the inset, and the initial force rate is derived by linear fitting.

The results of the force measurement tests are shown in Figure 7: for each elastomer (AT 31 F, Viton) and for each molarity (1 M, 2 M), the force averaged over the performed triplicate experiments is reported versus the time. In particular, the forces recorded during the first 3 min are shown, because we were specifically interested in the initial force rate. In consideration of the set-up used for such measurements (Figure 7A), we expected the force trend to be ruled by the sensor stiffness. Indeed, the sensor stiffness was much greater than that of the elastomeric disk for small bulging, independently of the disk material. The obtained results confirmed this expectation. It was straightforward to define an average force trend that depended only on the solute concentration (see the inset in Figure 7B). To quantitatively assess the measured behavior of the actuator, we considered the load cell as an external elastic load opposing bulge formation, so as to apply the model detailed in File S1, including Eq.5–12. Based, in particular, on Eq.7, the initial force rate should be proportional to the solute concentration; hence, we expected the ratio of the initial force slopes to be 2. We therefore estimated the initial force rate by linearly fitting the first 15 s of the measurements (based on the estimate in File S1, the associated error is around 1%), and we obtained 1.96, in excellent agreement with the model predictions. These results further corroborated our model-based approach used for designing the osmotic actuator.

For completeness, let us mention that the measured force increased during the observation time. For the AT 31 F elastomeric disk, it reached 15 N and 23 N for the 1 M and 2 M NaCl solutions, respectively. For the Viton disk, it reached 14 N and 20 N, respectively. These figures seemed to be consistent with the fact that Viton stiffening, as observed during the previous free bulge tests, bore an incrementally larger fraction of the osmotic pressure load. As anticipated, we were not specifically interested in measuring the maximum value of the actuation force, because it can be significantly affected by the specifically actuated load. However, the measured values suggested that our actuators can deliver a few Newtons on a minute timescale and tens of Newtons over several minutes.

## Discussion

We successfully designed and fabricated a forward osmosis-based actuator with a typical size of 10 mm and a characteristic time of 2–5 minutes, which can produce forces above 20 N and thus usable for several actuation tasks. Moreover, our actuator exhibits low power consumption, high energy density, and remarkable efficiency, as quantitatively detailed below.

**Figure 8:**
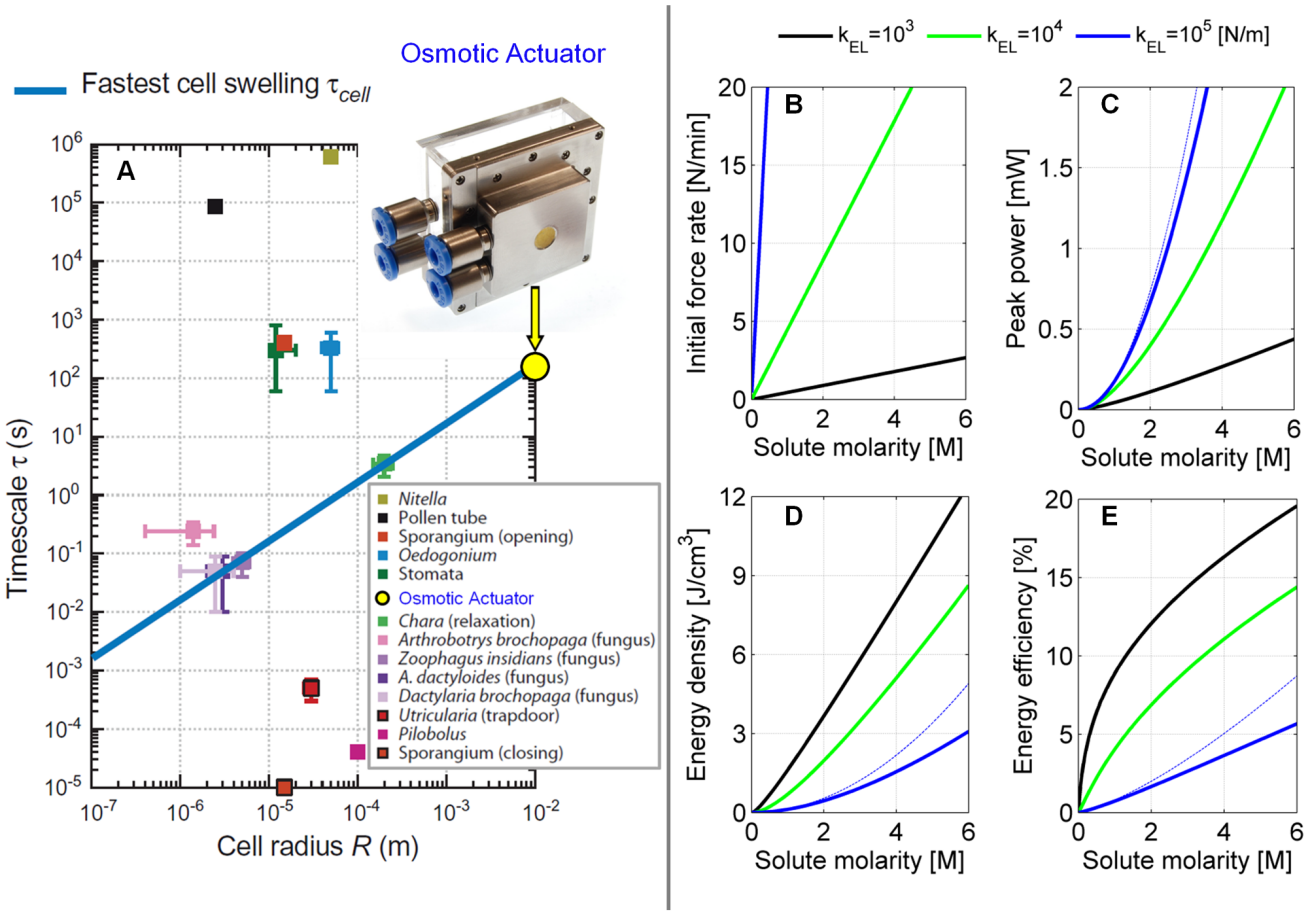
Osmotic actuator timescale and computed performance. (A) The osmotic actuator timescale 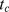 matches with the relaxation time of an ideal, giant plant cell with the same typical size, i.e. cell radius 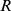. Adapted from [6] (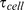 computed with 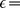
30 MPa and 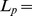
2·10^−12^ m s^−1^ Pa^−1^; the order of the labels in the figure key corresponds to their order in the figure from top to bottom). Computed performance of the osmotic actuator, coupled to an external elastic load with stiffness 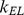 as a function of the NaCl concentration: (B) initial force rate 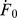; (C) peak power 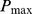; (D) energy density 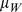; (E) thermodynamic energy efficiency 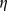. Solid curves were obtained from the corresponding expressions in File S1 (Eq.7, Eq.11, Eq.15, and Eq.16); the initial force rate is also reported in the present text (Eq.7). The following parameters were used: 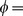
1, 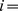
2, 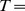
298K, 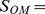
100 mm^2^, 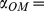
3·10^−13^ m s^−1^ Pa^−1^, 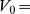
360 mm^3^, and 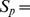
20 mm^2^. Dashed (thinner) curves were obtained from the associated approximate expressions (Eq.8, Eq.11, and Eq.12 in the present text), which more accurately apply for stiffer loads.

As regards timescale, our actuator is the fastest osmotic actuator developed so far [25], [29]. Moreover, Figure 8A highlights that the achieved timescale is very close to the relaxation time of an ideal, giant plant cell with the same typical size as the actuator. Furthermore, the actuator timescale is not far from the one of typical plant cells, such as the stomata shown in Figure 8A. Indeed, it is remarkable how our device and actual plant cells can get to comparable timescales starting from different values of the involved physical quantities, as reported in Table 2. In the considered table, the actuator volumetric stiffness is associated with the hemispherical bulging; based on the estimate of 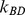, such a stiffness can be approximated as 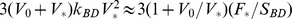, with 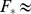
14–19 N for the considered elastomers (see File S2 for additional details). It should be noted that the resulting actuator volumetric stiffness is very close to a typical cell bulk modulus, e.g. 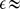
30 MPa [6]. These considerations fully support the pursued bioinspired approach.

**Table 2:** Main physical properties of a typical plant cell, and of the osmotic actuator.

	Typical size [m]	Compliant boundary (a) thickness [m]	Compliant boundary (a) Young modulus [MPa]	Membrane (b) permeability [m s^−1^ Pa^−1^]	Osmolyte concentration [M]	Volumetric stiffness (c) [MPa]
Plant cell	10^−5^–10^−4^ [4], [21] (a)	10^−7^–10^−6^ [37]	≈10^3^ [38]	10^−13^–10^−11^ [4]	0.5 (KCl), 1 (sucrose ) [39]	1–50 [4]
Osmotic actuator	10^−2^	10^−3^	≈10 (AT 31 F), 10^2^ (Viton) (d)	3·10^−13^	1–6 (NaCl)	≈25 (AT 31 F), 35 (Viton) (e)

(a) Cell membrane for the plant cell, bulging disk for the actuator.

(b) Cell membrane for the plant cell, osmotic membrane for the actuator.

(c) Cell bulk modulus for the plant cell, volumetric stiffness for the actuator.

(d) See File S3.

(e) See File S2.

Based on the confidence we gained in the underlying modeling approach, let us compute the actuator performance, e.g. in the case of an external elastic load. This is consistent with many implementations, such as any triggering mechanisms to be osmotically released, also in analogy to the stimulus-motion phase of the above described plant movements. Let us then assume a stiffness 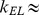
10^3^–10^5^ N m^−1^ for the external load; lower values are consistent e.g. with the stiffness of a steel helical spring having a winding diameter of the coil that fits with 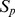 as in Figure 2A, while higher values are considered for the sake of illustration. Selected performance trends are then shown in Figure 8B–E, as a function of the NaCl initial concentration. These curves practically span the whole solubility range, since the maximum achievable NaCl concentration is 6.14 M (brine). The force rate trend shows that the osmotic actuator can provide forces well above 10 N in a few minutes (as experimentally recorded). Moreover, low power consumption is obtained across the whole solubility range (the peak power is on the order of 10^−1^–1 mW), as targeted, while the corresponding energy density is on the order of 1–10 J cm^−3^. Finally, thermodynamic efficiencies up to 20% can be achieved; in particular, efficiencies above 10% can be reached already at 2 M when the external load is not too stiff. Further performance estimates as a function of the actuation chamber volume or the external load stiffness can be easily obtained from the scaling laws derived in File S1; quick estimates can be also obtained through the approximate expressions in Eq.5–12. For instance, both the energy density and efficiency are expected to increase when the actuation chamber volume is decreased.

It is worth noting that, based on the computed performance, the osmotic actuator can challenge a muscle-based actuation strategy, at least at the considered lengthscale. Indeed, even if the efficiency of the mammalian skeletal muscle reaches 40% [40], its output power at the cubic centimeter scale is approximately 0.2 mW, while its corresponding maximum force and energy density are approximately 0.35 N and 10^−2^–10^−1^ J cm^−3^, respectively [40]. Moreover, despite its potential advantages, including the fact that the working mechanism of muscle is well understood and that its performances are largely scale-invariant [41], it is very challenging to effectively resort to muscle-based, hybrid actuation systems [42]. Conversely, the osmotic actuator bypasses the complexity of actin-myosin-like structures by simply exploiting the osmotic potential in a suitably designed actuation chamber, in close analogy to the plant model. Furthermore, the bulk materials of our osmotic actuator fulfill the molecular simplicity criterion and do not lead to significant temperature variations during activation, which can be detrimental for performance.

Moreover, in order to further assess the application potential of the osmotic actuator, let us consider its performance in relation to that of existing actuation technologies that could be implemented on the same lengthscale. Relevant performance indicators are reported in Table 3, where the osmotic actuator aims to compete with low-power-consumption technologies. However, a direct comparison amongst the considered indicators might be prone to inaccuracies, since their definition is often loosely reported in the cited references (a typical case is that of energy efficiency), and they are often evaluated in a broad set of working conditions. In particular, it is not straightforward to account in a comprehensive manner for supplementary energy expenditures needed to actually operate the considered actuators. For instance, electric energy is needed to simply keep the shape of conductive polymer-based actuators, while pumping work is required to operate pneumatic actuators as well as for the remote operation of the osmotic actuator, namely to load/flush the actuation chamber. Nevertheless, the proposed forward osmosis-based actuation strategy might provide a means for simplification with respect to other technologies, since the actuation power is generated by the mere contact between two solutions.

**Table 3:** Actuation performance indicators: a comparison of the osmotic actuator to existing actuation technologies.

Actuator Category	Actuator Subcategory	Output Power [mW]	Max. Force [N]	Energy Efficiency [%]	Energy Density [J cm^−3^]	References
Osmotic	Osmotic (Forward Osmosis)	10^−1^–1(a)	>10(b)	4–20^(a,c)^	1–10(a)	[this work]
Fluid-based	Hydraulic	10^2^	10^3^	90–99	10	[43], [44]
	Pneumatic	10^−1^–10^2^	10	30–40	10^−1^	[43], [45], [46]
Piezoelectric	Piezoelectric Ceramic	10–10^3^	10	30	10^−1^	[47], [48]
Shape Memory Alloys (SMA)	Thermally Activated SMA	10^−2^	10^−1^	0.01	>10^2^	[49]–[52]
	Ferromagnetic SMA	10^2^	10^2^	10	10^−3^–10^−1^	[53], [54]
Magnetostrictive	Magnetostrictive	10^2^	10^2^–10^3^	80–99	10^−2^	[55]–[57]
Nanostructures-based	Carbon Nanotubes aerogel	10	10^−3^	40	10^−3^–10^−2^	[58], [59]
Electroactive polymers	Dielectric Elastomers	1	1	50(c)	10^−1^–1	[43], [53], [60]–[62]
	Electrostrictive Polymers	10	10^−1^	30–50	10^−1^–1	[63]–[66]
	Conducting Polymers	10^−2^–1	1	0.25	10^−1^–1	[67]–[69]
	Ionic Polymer Metal Composites	10^2^–10^3^	10^−1^	2–3(c)	10^−3^–10^−2^	[70]–[72]
	Piezoelectric polymers	10	10^−2^	90–95	10^−3^	[53], [62], [73]
	Liquid Crystal Elastomers	10^2^	10	<5	10^−3^–10^−1^	[53], [62], [74]
	Shape Memory Polymers	10–10^2^	10	2–8	1	[75], [76]

(a) Exemplificative figures based on the computed trends in Figure 8C-E for 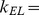
10^3^ and 10^4^ N m^−1^.

(b) Forces above 20 N were measured for a 2 M NaCl water solution within 10 min actuation times.

(c) Thermodynamic efficiency.

However, several technical aspects must be further addressed to completely characterize the osmotic actuation strategy. For instance, the behavior of the osmotic membrane under high pressures and solute concentrations should be better assessed, as for mechanical damage, permeability degradation, and membrane polarization effects. Furthermore, chemical instabilities potentially arising in concentrated solutions, which could produce solute precipitation and subsequent performance degradation, also must be assessed. In addition, further miniaturization is not trivial, and leakage prevention must be systematically achieved, such that the effective integration of the proposed osmotic actuator in a miniature device might pose further issues. Finally, we are aware of the fact that the present design can be hardly compared to reversible actuation systems, and we make no strong claims on regard. However, we beg to remark that the osmosis-based device described in [25] is not reversible (while reversibility is only mentioned in the patents [30], [31], whose concept has not been implemented so far). Nonetheless, a first step towards multiple actuations could be achieved by depleting the initial osmotic potential through successive steps. In particular, a predefined number of actuation shots could be achieved by designing the system in such a way that each shot is also used to set the osmotic potential for the next one. Anyway, it seems reasonable to speculate on a more refined embodiment of the proposed osmotic actuation in light of specific applications, and this will be addressed in a subsequent study.

Nevertheless, in light of the achievements of the present work, the osmotic actuator has potential impact on many fields, including biorobotics. Finally, let us emphasize that such a novel actuator stems from our model-based and bioinspired approach. On the one hand, in fact, we properly encompassed key physical aspects of water-driven actuation in plants; on the other hand, we pursued an integrated strategy by concurrently addressing and solving design and material issues. Overall, such an approach proved to be successful in extracting smart cues from the Plant Kingdom.

## Supporting Information

File S1 **Actuator performance indicators, in the presence of an external elastic load.** Simple analytical expressions were derived to describe the dynamic performance of the actuator when coupled with an elastic external load. These expressions were used, in particular, to interpret the measured force rates and to predict the relevant performance trends over the full NaCl solubility range.
(PDF)

File S2 **Free bulging dynamics of the osmotic actuator.** A simple theoretical model was introduced for describing the free bulge dynamics; model predictions well matched the experimental observations. Simplified analytical expressions were also introduced to describe the dynamic performance of the actuator, including in particular the timescale, for small bulging deformations.
(PDF)

File S3 **Osmotic actuator design and fabrication.** Actuator design and fabrication were detailed, by also discussing the choice of relevant components (osmolyte, structural material, osmotic membrane, and elastomers for the bulging disk).
(PDF)

Video S1 **Osmotic actuator free bulging.** After providing a schematic representation of the main components and assembly of the osmotic actuator, this video shows the free bulging deformation of the actuator elastomeric disk, as obtained for a 2 M NaCl solution. The characteristic time for such an actuation is 2 min.
(MP4)
