# Supplementary material for: Another Lesson from Plants: The Forward Osmosis-Based Actuator
Source: PLoS One. 2014 Jul 14;9(7):e102461. doi: 10.1371/journal.pone.0102461 (PMC4097062; doi:10.1371/journal.pone.0102461)
Supplement: File S1 — Actuator performance indicators, in the presence of an external elastic load. Simple analytical expressions were derived to describe the dynamic performance of the actuator when coupled with an elastic external load. These expressions were used, in particular, to interpret the measured force rates and to predict the relevant performance trends over the full NaCl solubility range. (PDF) [file pone.0102461.s001.pdf]

Supporting Information S1: Actuator performance, in the presence of an external elastic load

Let us preliminary recall relevant results from [1], where a simple model was introduced for describing the dynamic performance of the envisaged osmotic actuator. A two-chamber system was considered (see Figure 1), in which solvent flows from the Reservoir Chamber (RC) to the Actuation Chamber (AC) through an Osmotic Membrane (OM) having surface area S_{OM} and permeability α_{OM}. Moreover, the osmotic actuation work is gathered through the elastic deformation of an external load, namely a piston with surface area S_p and stiffness k_{EL}, whose displacement is denoted by δ. At the initial time, the AC volume is V_0, and it contains a molar concentration of the osmotic agent such that the corresponding osmotic pressure (driving the solvent flow) is Π_0. Further details can be found in [1].

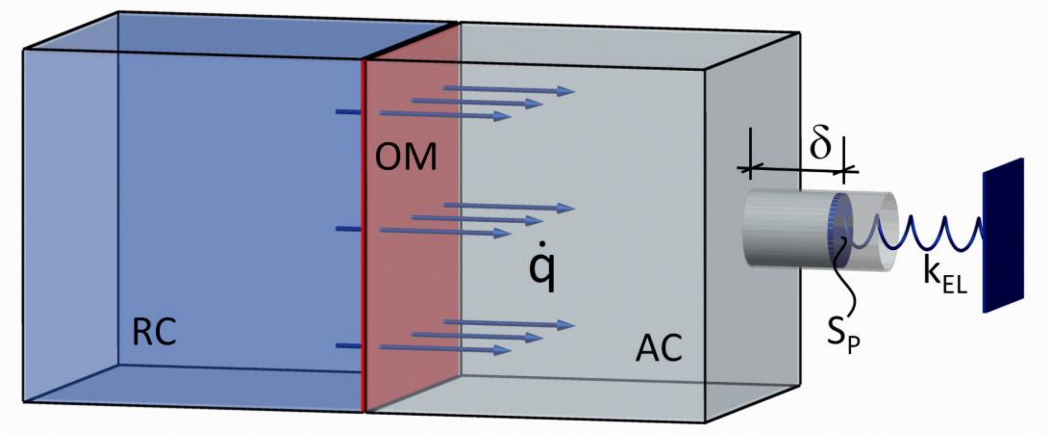

Figure 1. Schematic of the osmotic actuator also showing the solvent flux ($\dot{q}$) through the osmotic membrane. Actuation work is gathered through the displacement of an external elastic load, namely a piston.

The following expression was obtained for the evolution of the AC volume V versus time t:

$$2\frac{t}{\bar{t}} = \log_e\left[\frac{\Psi(1;C)}{\Psi(v;C)}\right], \tag{1}$$

where

$$\Psi(\xi;C) := \left(v_1^* - \xi\right)^{1+1/\omega}\left(\xi - v_2^*\right)^{1-1/\omega}, \tag{2}$$

with $v := V/V_0$, $\bar{t} := S_p^2/(k_{EL}S_{OM}\alpha_{OM})$, $C := \Pi_0 S_p^2/(V_0 k_{EL})$, $\omega := \sqrt{1+4C}$, $v_1^* := (1+\omega)/2$ and $v_2^* := (1-\omega)/2$. In particular, the non-dimensional AC volume monotonically increases from the initial value (equal to 1), and it asymptotically tends to the regime value $v_\infty = v_1^*$. For completeness, we recall that such a solution was obtained by integrating the following differential problem:

$$\frac{dv}{d\tau} = C\frac{1}{v} - (v-1),\ v(0) = 1, \tag{3}$$

where $\tau := t/\bar{t}$.

Based on the solution provided by Eq.1-2, the piston approaches the regime state on a timescale t_c defined as follows:

$$t_c = \frac{\bar{t}}{2} = \frac{S_p^2}{2k_{EL} S_{OM} \alpha_{OM}} . \tag{4}$$

Moreover, the actuation force reads:

$$F = k_{EL}\delta = k_{EL} \frac{V - V_0}{S_p} = \frac{k_{EL}V_0}{S_p}(v-1), \tag{5}$$

whence the maximum force (which is obtained in correspondence of the maximum piston displacement) is

$$F_{\max} = \frac{k_{EL}V_0}{S_p}(v_\infty - 1) = \frac{k_{EL}V_0}{S_p}\left(\frac{\sqrt{1+4C}-1}{2}\right). \tag{6}$$

It should be noticed that the force trend is proportional to the one of the volume increment. From Eq.5 and Eq.3 we immediately get the initial slope:

$$\dot{F}_0 := \left.\frac{dF}{dt}\right|_{t=0} = \frac{k_{EL}V_0}{\bar{t}S_p}\left.\frac{dv}{d\tau}\right|_{\tau=0} = \frac{k_{EL}V_0}{\bar{t}S_p} C = \frac{k_{EL}\Pi_0 S_{OM}\alpha_{OM}}{S_p} . \tag{7}$$

Moreover, by recalling the relevant expressions, the actuation power reads:

$$P = \frac{k_{EL}\delta}{S_p}\frac{dV}{dt} = \frac{k_{EL}V_0}{\bar{t}S_p}\frac{V - V_0}{S_p}\frac{dv}{d\tau} = \frac{k_{EL}V_0^2}{\bar{t}S_p^2}(v-1)\left(C\frac{1}{v} - (v-1)\right). \tag{8}$$

Clearly, $P(t=0) = P(t \to \infty) = 0$, as expected. Furthermore, by differentiating with respect to v we get:

$$\frac{dP}{dv} = \frac{k_{EL}V_0^2}{\bar{t}S_p^2}\left(C\frac{1}{v^2} - 2(v-1)\right). \tag{9}$$

From Eq.9 it is evident that $\frac{dP}{dv}(v=1) = \frac{k_{EL}V_0^2}{\bar{t}S_p^2} C > 0$, so that P initially increases. In addition, $\frac{dP}{dv}(v = v_\infty) = \frac{k_{EL}V_0^2}{\bar{t}S_p^2 v_\infty^2}\left(C - 2\left(v_\infty^3 - v_\infty^2\right)\right)$. Yet $v_\infty^3 - v_\infty^2 = Cv_\infty$ (since $v_\infty^2 - v_\infty - C = 0$, cf. Eq.3), and therefore $\frac{dP}{dv}(v = v_\infty) = \frac{k_{EL}V_0^2 C}{\bar{t}S_p^2 v_\infty^2}(1 - 2v_\infty) < 0$. Hence, there is a maximum $P_{\max}$ during the actuation evolution; it occurs in correspondence of a volume $v_{P_{\max}}$ which satisfies the equation $v^3 - v^2 - C/2 = 0$. Standard algebraic manipulations show that the sough value reads:

$$v_{P_{\max}} = \frac{\varphi + 1 + \varphi^{-1}}{3}\text{, where } \varphi = \varphi(C) := \left[\frac{2 + 27\tilde{C} - 3\sqrt{3}\sqrt{27\tilde{C}^2 + 4\tilde{C}}}{2}\right]^{1/3}\text{, where } \tilde{C} := C/2\text{,} \quad (10)$$

so that the corresponding peak power is:

$$P_{\max} = \frac{k_{EL}V_0^2}{\bar{t}S_p^2}\omega(C)\text{, where } \omega(C) := C\frac{\varphi - 2 + \varphi^{-1}}{\varphi + 1 + \varphi^{-1}} - \frac{(\varphi - 2 + \varphi^{-1})^2}{9}. \quad (11)$$

Then, power density is defined as follows:

$$\mu_P := \frac{P_{\max}}{V_0}. \quad (12)$$

Furthermore, the elementary actuation work is given by

$$dW = \frac{k_{EL}\delta}{S_p}dV = \frac{k_{EL}V_0}{S_p}\frac{V - V_0}{S_p}dv = \frac{k_{EL}V_0^2}{S_p^2}(v - 1)dv, \quad (13)$$

whence the actuation work (up to regime) reads

$$W = \frac{k_{EL}V_0^2}{S_p^2}\frac{(v_\infty - 1)^2}{2} = \frac{k_{EL}V_0^2}{S_p^2}\sigma(C)\text{, with } \sigma(C) := \frac{1 + 2C - \sqrt{1 + 4C}}{4}. \quad (14)$$

Moreover, energy density is defined as follows:

$$\mu_W := \frac{W}{V_0}. \quad (15)$$

Finally, thermodynamic energy efficiency is defined as follows [2]:

$$\eta := \frac{W}{W_{\max}} = \frac{W}{|\Delta A|} = \frac{W}{|\Delta G - W_{\exp}|} = \frac{W}{|\Delta G - W|}, \quad (16)$$

where the maximum work $W_{\max}$ can be derived from the Helmholtz free-energy variation ΔA (final minus initial), which in turn can be expressed in terms of the Gibbs free-energy variation ΔG and the expansion work $W_{\exp}$. For the present case, latter term is suitably provided by the actuation work, so that we obtain the last equality in Eq.16. Moreover, ΔG is here related to the mixing between the NaCl solution and the pure water entering the actuation chamber through the osmotic membrane. A relevant expression is [3]

$$\Delta G = \Delta G_{mix} = nRT\left(x_s \ln x_s^i + x_w \ln x_w\right), \quad (17)$$

where x_s and x_w respectively denote the molar fraction of salt and water ($x_s + x_w = 1$), and the Van't Hoff coefficient i accounts for sodium chloride dissociation in water. As we deal with dilute solutions, it is $x_w \ln x_w \cong 0$, and we can simplify Eq.17 into $\Delta G_{mix} \cong n_s RTi \ln x_s$, where $n_s := nx_s$. Moreover, since $\Pi_0 \cong iM_0 RT = i(n_s/V_0)RT$, we get $\Delta G_{mix} \cong \Pi_0 V_0 \ln x_s$ and we finally obtain the following expression for the efficiency:

$$\eta \cong \frac{W}{|\Pi_0 V_0 \ln x_s - W|} . \qquad (18)$$

The obtained expressions can be partly simplified in the case of stiffer external loads. We hereafter provide such simplifications, for completeness, and also because they can be used to easily draw some considerations on the actuator performance scaling. For the sake of definiteness, let us consider some reference values for the relevant actuation parameters, consistently with the main data already introduced in the paper. In particular, let us assume to use a 1 M NaCl solution, so that we have $\Pi_0 \cong$ 5 MPa. Moreover, we consider a 10x10 mm osmotic membrane, and thus $S_{OM} = 10^{-4}$ m^2, with permeability $\alpha_{OM} \cong 3 \cdot 10^{-13}$ m s^{-1} Pa^{-1}. Moreover, let us choose $S_p / S_{OM} = 0.2$, so that $S_p = 2 \cdot 10^{-5}$ m^2. Hence, for t_c not to exceed, say, 10 min, we should choose $k_{EL} \geq 10^4$ N m^{-1}. Then, by assuming a 12x12x2.5 mm actuation chamber, so that $V_0 = 3.6 \cdot 10^{-7}$ m^3, we get that $C \cong 5 \cdot 10^3 / k_{EL} \leq 0.2$. Therefore, for the working range of our interest, we can assume that C is small-enough (ideally $C << 1$) to allow for some simplification, by Taylor-expansion, of the above expression regarding power and actuation work. In particular, we obtain

$$\frac{\sqrt{1+4C}-1}{2} \approx C , \qquad (19)$$

$$\omega(C) \cong \frac{1}{4}C^2 - \frac{1}{4}C^3 + \frac{3}{8}C^4 - \frac{11}{16}C^5 + O(C^6) \approx \frac{1}{4}C^2 \text{, and} \qquad (20)$$

$$\sigma(C) \cong \frac{1}{2}C^2 - C^3 + \frac{5}{2}C^4 - 7C^5 + O(C^6) \approx \frac{1}{2}C^2 , \qquad (21)$$

so that, by only retaining the leading terms, we get to the following approximations:

$$F_{\max} \approx \frac{k_{EL} V_0}{S_p} C = \frac{k_{EL} V_0}{S_p} \frac{\Pi_0 S_p^2}{V_0 k_{EL}} = \Pi_0 S_p , \qquad (22)$$

$$P_{\max} \approx \frac{1}{4} \frac{k_{EL} V_0^2}{\bar{t} S_p^2} C^2 = \frac{1}{4} \frac{k_{EL}^2 V_0^2 S_{OM} \alpha_{OM}}{S_p^4} \frac{\Pi_0^2 S_p^4}{k_{EL}^2 V_0^2} = \frac{S_{OM} \alpha_{OM} \Pi_0^2}{4} , \qquad (23)$$

$$\mu_P \approx \frac{S_{OM} \alpha_{OM} \Pi_0^2}{4 V_0} , \qquad (24)$$

$$W \approx \frac{1}{2}\frac{k_{EL}V_0^2}{S_p^2}C^2 = \frac{1}{2}\frac{k_{EL}V_0^2}{S_p^2}\frac{\Pi_0^2 S_p^4}{V_0^2 k_{EL}^2} = \frac{S_p^2\Pi_0^2}{2k_{EL}}, \quad (25)$$

$$\mu_W \approx \frac{S_p^2\Pi_0^2}{2k_{EL}V_0}, \quad (26)$$

$$\eta \approx \frac{\frac{S_p^2\Pi_0^2}{2k_{EL}}}{\Pi_0 V_0 |\ln x_s| + \frac{S_p^2\Pi_0^2}{2k_{EL}}} = \frac{1}{1+\frac{2k_{EL}V_0|\ln x_s|}{S_p^2\Pi_0}}. \quad (27)$$

As anticipated, the obtained simplified expressions permit to quickly draw some comments on the actuator performance. For instance, for stiff-enough external loads ($C << 1$) we get $P_{\max} \approx 0.2$ mW independently of the external load; this exemplificative value confirms the very low power consumption of the envisaged osmotic actuator. Moreover, it is worth noticing how the efficiency increases by reducing V_0 or by increasing the osmotic potential Π_0.

The expressions provided by Eq.4, Eq.7, Eq.22, and Eq.23-27 are reported in the main text of the present paper (Table 1), for ease of presentation. Furthermore, some trends for Eq.7, Eq.11, and Eq.15-16 are also shown (Figure 9).

A remark on the estimate of the initial force rate

When discussing the actuator force rate measurement in the main text, we estimated the initial rate $\dot{F}_0$ by considering the first 15 seconds of the measurements. Such a choice is better documented hereafter.

In light of the experimental set-up used for the force measurement, bulging was opposed by the internal action of the elastomeric disk itself, and by the external reaction of the load-cell sensor. The latter force was approximated as an external elastic load, of the type of that one introduced above. Indeed, based on the load-cell datasheet, it can produce a reaction force up to 500 N in correspondence of a displacement as small as roughly 15 μm, and it is reasonable to assume a linear constitutive relation over such a small deformation (after all, the load-cell sensor is a rather standard mechanical component). Furthermore, in correspondence of such a very small displacement, the internal force which opposing the bulge is negligible (cf. relevant data in Supporting Information S2). Hence, we were encouraged to use the piston model introduced above in order to interpret the force measurement results. The fact that the measured force trends (reported in Figure 2) did not depend, in practice, on the specific elastomer confirmed the above position, thus supporting the exploitation of the piston model.

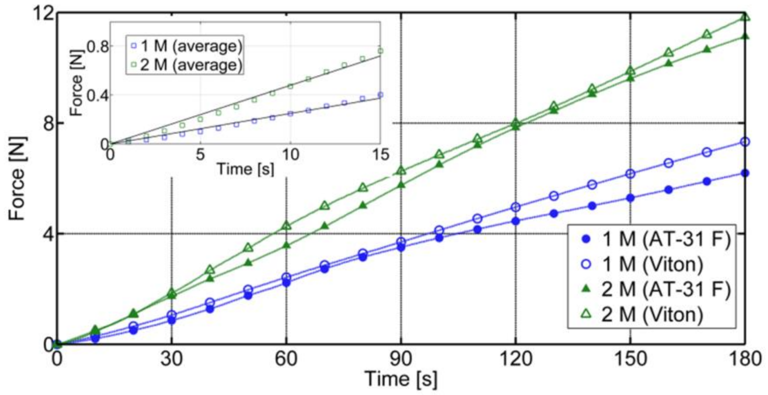

Figure 2. Measured actuator force: data points represent averages over the triplicate tests. (This figure replicates Figure 8B of the main text; it is reported here for ease of presentation.)

We firstly observed that at $t=0$ we have from Eq.3 $dv/d\tau = C$. Still from the same relation we get that a small relative slope decrement, say $dv/d\tau = C(1-\varepsilon)$ with $\varepsilon << 1$, is obtained in correspondence of a small volume variation $\varsigma = v - 1 << 1$ such that, at a first-order approximation, $\varsigma \approx C\varepsilon/(1+C)$ (obviously, ε must not be confused with the cell bulk modulus introduced in the main text). Moreover, the decremented slope is taken at a time t_ε which can still be estimated through Eq.3, namely:

$$\frac{t_\varepsilon}{\bar{t}} \approx \frac{\varepsilon}{1+C}. \tag{28}$$

We then observed that, from Eq.7 and from the relevant definitions, the following relations hold:

$$\bar{t} = \frac{\Pi_0 S_p}{\dot{F}_0}, \tag{29}$$

$$C = \frac{\Pi_0^2 S_p S_{OM} \alpha_{OM}}{V_0 \dot{F}_0}. \tag{30}$$

Finally, we observed that an estimate $\dot{F}_\varepsilon$ of the initial slope $\dot{F}_0$ could be obtained through a standard linear fitting of the force measurements over an interval $[0, t_\varepsilon]$. In this regard, t_ε must be properly chosen: on the one hand it must be large enough to contain a significant number of measures, on the other hand it must be not too large, so as to properly approximate the initial slope. An estimate of the error which is introduced when adopting $\dot{F}_\varepsilon$ can be achieved by plugging $\dot{F}_\varepsilon$ into Eq.29-30 in place of $\dot{F}_0$, and by substituting such expressions into Eq.28 so as to finally obtain ε. Formally, we get:

$$\varepsilon \approx \frac{t_\varepsilon}{\bar{t}(\dot{F}_\varepsilon)}\left(1 + C(\dot{F}_\varepsilon)\right) = \frac{t_\varepsilon \dot{F}_\varepsilon}{\Pi_0 S_p}\left(1 + \frac{\Pi_0^2 S_p S_{OM} \alpha_{OM}}{V_0 \dot{F}_\varepsilon}\right). \quad (31)$$

We then recalled our parameters: S_{OM} = 10^{-4} m^2, $\alpha_{OM} \cong 3 \cdot 10^{-13}$ m s^{-1} Pa^{-1}, S_p = $2 \cdot 10^{-5}$ m^2, and $V_0 = 3.6 \cdot 10^{-7}$ m^3. Moreover, we considered the experiments with 1 M NaCl solution (so that $\Pi_0 \cong$ 5 MPa); and we fitted the averaged data (averaged over the two considered elastomers). In particular, we chose $t_\varepsilon = 15$ s, so as to consider 15 experimental points (indeed, force acquisition rate was 1 s^{-1}). From the linear fitting we obtained $\dot{F}_\varepsilon \approx 2.5 \cdot 10^{-2}$ N s^{-1} ($R^2 = 0.99$), and from Eq.31 the estimated error was $\varepsilon \approx 10^{-2}$, thus surely acceptable for our purposes. Furthermore, by repeating this procedure by varying t_ε in the interval [10,20] s, we assessed $\dot{F}_\varepsilon \approx (2.5 \pm 0.1) \cdot 10^{-2}$ N s^{-1} (mean ±std), so that the chosen time span t_ε seemed adequate for the sought estimate. We also performed the above mentioned procedure for the 2 M NaCl solution, thus obtaining $\dot{F}_\varepsilon \approx 4.8 \cdot 10^{-2}$ N s^{-1} ($R^2 = 0.99$), with $\varepsilon \approx 1.6 \cdot 10^{-2}$, in correspondence of $t_\varepsilon = 15$ s, and $\dot{F}_\varepsilon \approx (4.8 \pm 0.2) \cdot 10^{-2}$ N s^{-1} (mean ± std) over the [10,20] s interval for t_ε. In light of these figures, the initial force slope was suitably assessed. Incidentally, by using Eq.7 together with the obtained slope, we estimated that the actuator perceived an external stiffness around $3.3 \cdot 10^3$ N m^{-1} (such an apparent stiffness also depends on the actual contact between the load cell sensor and the elastomeric disk; for instance, no lateral confinement was enforced for the latter).

To conclude, based on the (mean ± std) slope figures, the resulting ratio between the two slopes (2 M over 1 M) was 1.96±0.16. This is in excellent agreement with the adopted model of the actuator, according to which the considered ratio should be ideally 2. This result, in particular, confirms that in the presence of an external elastic load, the initial slope $\dot{F}_0$ of the actuator linearly scales with Π_0, as predicted by Eq.7.

References

1. Sinibaldi E, Puleo GL, Mattioli F, Mattoli V, Di Michele F, Beccai L, Tramacere F, Mancuso S, Mazzolai B (2013) Osmotic actuation modeling for innovative biorobotic solutions inspired by Plant Kingdom. Bioinspiration & Biomimetics, 8: 025002.
2. Atkins P, De Paula J (2006) Atkins' Physical Chemistry. Oxford, UK: University Press.
3. Yip NY, Elimelech M (2012) Thermodynamic and Energy Efficiency Analysis of Power Generation from Natural Salinity Gradients by Pressure Retarded Osmosis. Environmental Science & Technology 46(9): 5230-9.
