# Supplementary material for: Another Lesson from Plants: The Forward Osmosis-Based Actuator
Source: PLoS One. 2014 Jul 14;9(7):e102461. doi: 10.1371/journal.pone.0102461 (PMC4097062; doi:10.1371/journal.pone.0102461)
Supplement: File S2 — Free bulging dynamics of the osmotic actuator. A simple theoretical model was introduced for describing the free bulge dynamics; model predictions well matched the experimental observations. Simplified analytical expressions were also introduced to describe the dynamic performance of the actuator, including in particular the timescale, for small bulging deformations. (PDF) [file pone.0102461.s002.pdf]

Supporting Information S2: Free bulging dynamics of the osmotic actuator

We hereafter elaborate the model introduced in [1], in order to describe the dynamics of the osmotic actuator, with reference to the free bulging experiments described in the main text of the present study. With reference to Figure 1, let $V_B(t) = V(t) - V_0$ denote the volume of the bulge, where $V(t)$ and V_0 respectively represent the volume of the actuation chamber (AC) at time t, and at the initial time $t = 0$ (subscript 0 is hereafter understood with this meaning). We also assume that there is no osmolyte rejection by the osmotic membrane (OM), which has surface area S_{OM} and permeability α_{OM}. Then, solvent mass conservation implies that:

$$\frac{dV_B}{dt} = \frac{dV}{dt} = \dot{q} = S_{OM}\alpha_{OM}\left(\Pi - \left(p - p_{RC}\right)\right), \qquad (1)$$

where p_{RC} denotes the pressure in the reservoir chamber (RC). For our purposes we can assume $p_{RC} \cong p_{ext}$ where p_{ext} denotes the external environment pressure. Moreover, by virtue of the classical Van't Hoff formula, we have $\Pi = \Pi_0 V_0 / V$. Furthermore, we assume a constitutive law for the bulging disk such that

$$p - p_{ext} = k_{BD} V_B^{\gamma}, \qquad (2)$$

where k_{BD} is a stiffness coefficient. For instance, for small bulging deformations $\gamma = 3$ (see [1] and references therein). In view of these positions, Eq.1 becomes:

$$\frac{dV_B}{dt} = S_{OM}\alpha_{OM}\left(\Pi_0 \frac{V_0}{V_0 + V_B} - k_{BD} V_B^{\gamma}\right). \qquad (3)$$

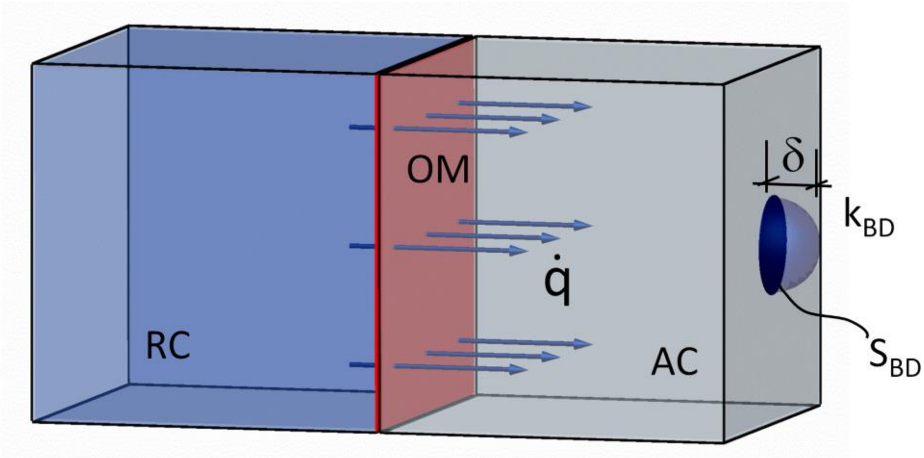

Figure 1. Schematic of the osmotic actuator also showing the solvent flux ($\dot{q}$) through the osmotic membrane. Actuation work is simply transduced into a bulging deformation of the elastomeric disk; bulge height is denoted by δ.

Let us then introduce a reference volume $V_* := 2\pi r^3/3$, where $r := \sqrt{S_{BD}/\pi}$ is the radius of the elastomeric disk, so that V_* is equal to the volume of a hemispherical bulge. Furthermore, let us introduce a reference time $t_* := V_*/\left(S_{OM}\alpha_{OM}\Pi_0\right)$.

In terms of the non-dimensional variables $v := V_B / V_*$ and $\tau := t / t_*$, it is immediate to recast Eq.3 as follows:

$$\frac{dv}{d\tau} = \left(\frac{\overline{V}_0}{\overline{V}_0 + v} - \frac{k_{BD} V_*^{\gamma}}{\Pi_0} v^{\gamma} \right), \tag{4}$$

with $\overline{V}_0 := V_0 / V_*$. Finally, by introducing the force exerted by the elastomeric disk in correspondence of a hemispherical bulge deformation, namely

$$F_* := S_{BD} k_{BD} V_*^{\gamma}, \tag{5}$$

we describe the bulge dynamics through the following differential problem:

$$\frac{dv}{d\tau} = \left(\frac{\overline{V}_0}{\overline{V}_0 + v} - \frac{F_*}{\Pi_0 S_{BD}} v^{\gamma} \right), \quad v(0) = 0. \tag{6}$$

As remarked in the main text, for our purposes it is of particular interest to describe the bulge dynamics up to the hemispherical shape, i.e. up to $v = 1$.

The model at hand only requires one calibration parameter, namely F_*. We measured this force by using a tabletop Instron™ equipment (Model 4464, ITW Test and Measurement, Italy S.r.l.) equipped with a hemispherical indenter; data were recorded by Labview™ 8.6 software (Labview Professional Development System, National Instrument, USA). It resulted F_*=14.0±0.1 N and F_*=19.0±0.8 N (mean±std over 5 measurements) for AT 31 F rubber and for Viton™, respectively. After calibration, Eq.6 was numerically integrated by using an adaptive, fourth-order accurate Runge-Kutta scheme [2], by choosing in particular $\gamma = 3$. Once obtained v, we derived the bulge height δ by approximating the bulge with a spherical cap, so that $V_B = \pi\delta(3r^2 + \delta^2)/6$. In practice, we solved the non-linear equation $v - x(3 + x^2)/4 = 0$ in order to obtain $x := \delta / r$, by using the Matlab™ (Mathworks, USA) software environment. Finally, we obtained the bulge protrusion height by subtracting 0.5 mm from the bulge height, in consideration of the fact that the elastomeric disk is glued onto an annular slot located 0.5 mm below the external surface of the actuator (see the AC design). The results of the numerical model Eq.6 are reported in Figure 2 (solid lines), together with experimental measures of the bulge protrusion height.

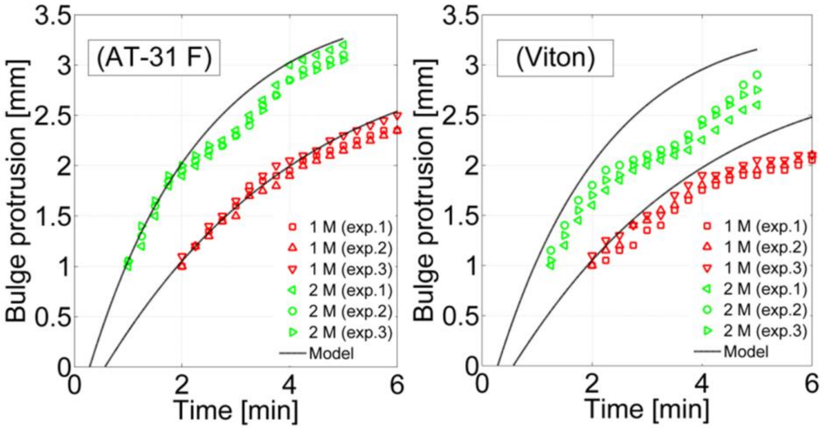

Figure 2. Bulge protrusion height versus time: experimental measures and theoretical model predictions. (This figure replicates Figure 7B of the main text; it is reported here for ease of presentation.)

Let us remark that we also verified that bulge dynamics up to hemispherical bulging is quite insensitive to variations of γ (indeed, the first part of the bulging dynamics is ruled by the contribution of the osmotic potential). Hence, our result turns out to be quite robust against potential uncertainties in the elastomer constitutive law. This, in turn, positively contributes to the physical representativeness of the model predictions.

Let us finally observe that, once measured F_* as described above, the stiffness of the considered elastomeric disks can be estimated from Eq.5 (with $\gamma = 3$), namely:

$$k_{BD} \approx \frac{F_*}{S_{BD} V_*^3} . \tag{7}$$

Such an estimate was actually performed in order to preliminarily assess the suitability of the chosen elastomers, as discussed in the main text and in Supporting Information S3. In particular, having chosen $r = 5$ mm, we obtained $k_{BD} \approx 2.03 \cdot 10^{28}$ and $2.8 \cdot 10^{28}$ N m^{-11}, for the AT 31 F rubber and for the Viton™ disk, respectively. Consistently, we can use Eq.7 to estimate the actuator volumetric stiffness $V(dp/dV)$ in correspondence of the hemispherical bulging configuration, which is a natural reference configuration for the case at hand. For such a configuration we have

$$\left(V_0 + V_*\right) \frac{dp}{dV}\bigg|_{V_0+V_*} \approx 3\left(V_0 + V_*\right) k_{BD} V_*^2 \approx 3\left(1 + \frac{V_0}{V_*}\right) \frac{F_*}{S_{BD}} . \tag{8}$$

It should be noticed how both the elastic and the geometrical properties of the compliant AC boundary, together with the AC size, affects the volumetric stiffness of the actuator. This is in full analogy with the plant cell model that has been taken as inspiration source for the present study. Indeed, the cell bulk modulus $\varepsilon = V(dp/dV)$, which characterizes how changes in cell volume are related to changes in turgor pressure, depends on both the elasticity of the cell wall and on the cell geometry [4]. From Eq.8 we get $V(dp/dV) \approx$ 25 and 35 MPa, for the AT 31 F rubber and for the Viton™ disk, respectively. These values are comparable to the bulk modulus of plant cells, which has a typical value between 1 and 50 MPa [4], $\varepsilon \approx$ 30 MPa being taken as a reference value [5]. This point further supports the bioinspired approach we pursued for developing the osmotic actuator.

A remark on the actuation performance in the case of small bulging deformations

The bulging of an elastic membrane can be described by the following relations [1,3]:

$$p - p_{ext} = k_{BD} V_B^3 , \tag{9}$$

with

$$k_{BD} = \frac{64\pi^2}{3} \frac{E}{1-\nu} \frac{t_h}{S_{BD}^5} , \tag{10}$$

where E, ν, and t_h respectively denote the Young modulus, the Poisson coefficient, and the thickness of the membrane. Eq.9-10 were considered in [1] (Eq.9 appears therein with a very minor notational difference), in order to study the bulging dynamics for the case of small deformations, i.e. for $V_B << V_0$. In particular, analytical expressions were obtained for the actuator performance indicators. It is worth remarking how Eq.9-10, which strictly apply in a linearly elastic framework, permit to obtain an explicit expression for the actuator volumetric stiffness in terms of the elastic (E, ν) and geometrical (t_h, S_{BD}) properties of the compliant AC boundary.

As shown in Figure 2, Eq.9 can be used to get an accurate description of the bulge dynamics, at least to hemispherical bulging, when also using an elastomeric disk as compliant AC boundary. Hence, we estimate below the performance indicators of the bulging osmotic actuator by adapting the corresponding expressions reported in [1]. We deliberately consider approximate expressions, for ease of presentation, being aware that they are more accurate in the limit of small bulging deformations.

The bulge asymptotically approaches a regime value, with the following timescale:

$$t_c \approx \frac{1}{3 S_{OM} \alpha_{OM} \Pi_0^{2/3} k_{BD}^{1/3}} , \tag{11}$$

whereas the maximum force and the initial force rate respectively read:

$$F_{\max} \approx \Pi_0 S_{BD} . \tag{12}$$

$$\dot{F}_0 \approx 0 . \tag{13}$$

Moreover, peak power, power density, actuation work (up the regime), and energy density are respectively given by the following expressions:

$$P_{\max} \approx \frac{S_{OM} \alpha_{OM} \Pi_0^2}{4} , \tag{14}$$

$$\mu_P \approx \frac{S_{OM} \alpha_{OM} \Pi_0^2}{4 V_0} , \tag{15}$$

$$W \approx \frac{\Pi_0^{4/3}}{4k_{BD}^{1/3}}, \tag{16}$$

$$\mu_W \approx \frac{\Pi_0^{4/3}}{4V_0 k_{BD}^{1/3}}. \tag{17}$$

Furthermore, by introducing the thermodynamic energy efficiency η as in Supporting Information S1, the following approximation is readily obtained:

$$\eta \cong \frac{W}{\left|\Pi_0 V_0 \ln x_s - W\right|} \approx \frac{1}{1+\frac{4k_{BD}^{1/3}V_0\left|\ln x_s\right|}{\Pi_0^{1/3}}}. \tag{18}$$

The expressions provided by Eq.11-18 are reported in the main text of the present paper (Table 1), for ease of presentation.

References

1. Sinibaldi E, Puleo GL, Mattioli F, Mattoli V, Di Michele F, Beccai L, Tramacere F, Mancuso S, Mazzolai B (2013) Osmotic actuation modeling for innovative biorobotic solutions inspired by Plant Kingdom. Bioinspiration & Biomimetics, 8: 025002.
2. Press WH, Teukolsky SA, Vetterling WT, Flannery BP (1992) Numerical recipes in C: the art of scientific computing 2nd edition. Cambridge, UK: University Press.

3 Timoshenko S, Woinowsky-Krieger S (1964) The theory of plates and shells 2nd edition. New York, US: McGraw-Hill

4 Dumais J, Forterre Y (2012) “Vegetable Dynamicks”: The Role of Water in Plant Movements. Annual Review in Fluids Mechanics 44: 453-78.

5 Forterre Y (2013) Slow, fast and furious: understanding the physics of plant movements. Journal of Experimental Botany 64(15): 4745-60.
