# Supplementary material for: Another Lesson from Plants: The Forward Osmosis-Based Actuator
Source: PLoS One. 2014 Jul 14;9(7):e102461. doi: 10.1371/journal.pone.0102461 (PMC4097062; doi:10.1371/journal.pone.0102461)
Supplement: File S3 — Osmotic actuator design and fabrication. Actuator design and fabrication were detailed, by also discussing the choice of relevant components (osmolyte, structural material, osmotic membrane, and elastomers for the bulging disk). (PDF) [file pone.0102461.s003.pdf]

Supporting Information S3: Osmotic actuator design and fabrication

We targeted a typical actuator size around 10 mm, based on preliminary estimates according to which this lengthscale should allow for characteristic actuation times on the order of a few minutes [1]. We preliminarily chose the working osmolyte, the structural material, and a commercially available osmotic membrane. We also fixed the surface area S_{OM} = 100 mm^2 of the osmotic membrane (OM), as well as a 5 mm diameter hole for the bulging disk protrusion. Afterwards, we assessed the suitability of a few selected elastomers for achieving a timescale on the order of a few minutes. The geometry of the actuation chamber (AC) was consistently defined, as well as the one of the reservoir chamber (RC). In particular, two modules were designed: one devoted to the RC, the other hosting the AC. The latter module included the OM and the elastomeric disk, which provided part of the AC boundary. Details for each of the aforementioned steps and associated fabrication issues are described below.

Osmolyte

Among the natural osmolytes exploited by plants, sodium chloride (NaCl) was an attractive candidate for our purpose, for several reasons. First, it shows nearly ideal osmotic behavior because it is perfectly dissociated in water, and this behavior is nicely described by Van't Hoff equation with $i = 2$ (with a 2% degree of tolerance) and $\phi \cong 1$ across the solubility range. Second, NaCl water solutions are indefinitely stable, and they permit osmotic pressure differences on the order of several MPa which, while being comparable to the biological systems that inspired this study, can also be effectively exploited for many actuation tasks. In addition, the manufacturing technology to produce forward (and reverse) osmotic membranes suitable for operation with NaCl is known, optimized and reproducible. Finally, NaCl is practical: it is inexpensive, easily available, and produces no dangerous waste.

Structural materials

Once we chose NaCl as osmolyte, we decided to use stainless steel as our structural material to minimize NaCl-promoted oxygen corrosion effects (namely changes in the ion concentrations, OM fouling, and insoluble material precipitation). In particular, AISI 316 (also called "marine grade") stainless steel was selected due to its enhanced corrosion resistance (achieved by adding molybdenum) in chloride-containing environments. Such a material was used for all the parts in contact with the osmotic solution, including the AC and the grids supporting the OM (see below). Moreover, AISI 316 was also exploited for the portion of the RC that was in contact with the AC, to facilitate the mechanical coupling/fastening of such two chambers. Indeed, corrosion in the RC should be negligible, provided that osmolyte spillage is minimized by a properly functioning OM and sealing elements. The remaining part of the RC was fabricated in Plexiglas, to allow for the visual inspection of the OM and the observation of actuator functioning.

Osmotic membrane

A semi-permeable forward-osmosis membrane specifically designed for operation with NaCl was purchased from HTI™ (Hydration Technology Innovations, Scottsdale, AZ, USA). The chosen membrane is made of cellulose triacetate cast onto a non-woven backing consisting of polyester fibers individually coated with polyethylene. Its water permeability coefficient is α_{OM} = $3 \cdot 10^{-13}$ m s^{-1} Pa^{-1}, its NaCl rejection coefficient σ is in the range 0.95-0.97 (thus close to the ideal case), and it permits very little NaCl counter-diffusion (approximately 0.5 g of NaCl for every liter of water crossing the OM). Moreover, this membrane has very low performance degradation due to NaCl fouling, making it a good technological solution that shows potentially ideal behavior.

Bulging elastomeric disk

It seemed suitable for the present scope to choose a commercially available elastomer, to ease actuator development. Hence, we selected the following elastomer polyisoprene composites: AT 31 F rubber, AT 84 CME rubber (SIGAP, Italy), Viton™ (Camthorne Industrial Suppliers, United Kingdom), EPDM Sh70, and EPDM Sh85 (Etter, Italy). Based on their Shore-A hardness, their corresponding values of Young modulus were expected to be in the range 10^7-10^8 Pa [2]. Moreover, we chose a 1 mm thickness for the disk. The mechanical behavior of the purchased elastomers was preliminarily assessed through standard stress-strain tests. In particular, ultimate tensile strength and material cyclic behavior were recorded using tabletop Instron™ equipment (Model 4464, ITW Test and Measurement Italy S.r.l.) with a load cell having 1 kN range. The Instron™ cross-head was slowly moved (1 mm/min), in view of the typical deformation speeds targeted for the considered elastomers. Test specimens were cut in bone shapes according to the ASTM D412-06a specifications (Standard Test Methods for Vulcanized Rubber and Thermoplastic Elastomers—Tension) and were carefully gripped so as not to induce parasitic tensions. All tests were repeated in triplicate, and the resulting data were recorded with Labview™ 8.6 software (Labview Professional Development System, National Instrument, TX, Austin) and processed in the Matlab™ (Mathworks, USA) software environment. Only the AT 31 F rubber, the AT 84 CME rubber, and the Viton™ elastomer performed well during the preliminary stress-strain tests, while the others exhibited highly inelastic behavior (even at low nominal strains) and low strength (with max tensile load of less than 25 N), so that they were discarded. The three selected elastomeric sheets were subsequently preconditioned through 10 cycles (mono-axial load, 2 orthogonal load directions) before laser-cutting disks with a 10 mm diameter (the outer circular region of the disk was glued to the micro-milled body of the AC module). Afterwards, also the AT 84 CME elastomer was discarded, since it suffered from water leakage during preliminary bulging tests. Finally, the potential for effective exploitation of the remaining elastomers, i.e. AT 31 F rubber and Viton™, was assessed in view of the actuation timescale (obtained in Supporting Information S2 and reported in the main text), which is here recalled for ease of presentation:

$$t_c \approx \frac{1}{3\,S_{OM}\,\alpha_{OM}\,\Pi_0^{2/3}\,k_{BD}^{1/3}}\,. \qquad (1)$$

In more detail, the stiffness k_{BD} was firstly evaluated through an indentation test, as described in Supporting Information S2. Then, by considering a 1 M NaCl solution (i.e. an initial osmotic potential $\Pi_0 \cong$ 5 MPa), the actuation timescale was estimated by using Eq.1: for both elastomers it turned out to be close to 2 min (more precisely, 2.3 min for the AT 31 F rubber, 2.1 min for Viton™). This result encouraged to actually fabricate the actuator by adopting both AT 31 F rubber and Viton™ elastomeric disks.

Actuation chamber module design

Two supporting grids were initially designed, aimed at sandwiching the OM to contain its deflection following exposure to the pressure increase in the AC. Indeed, an uncontrolled OM deflection can cause considerable performance loss [1]. The sought passage area for the water flux was then obtained by 16 square windows, each with a side length of 2.5 mm and separated by 0.5 mm wide ribs (Figure 1C). Moreover, the grid thickness was set to 0.25 mm to produce a maximum OM deflection below 0.1 mm, corresponding to a 1 MPa pressure difference. Such a deflection was estimated using finite element analysis software Abaqus 6.11 (Dassault Systèmes, France) by considering a 21x21x0.5 mm plate (see Figure 1C,E) that was fixed relative to the screw head contact surfaces. Here, the OM coincided with the mid-plane of the plate. A 12x12x2.5 mm working volume was then defined for the AC (Figure 1A,D). Its thickness was defined to allow the machining of 2 channels (see Figure 1A,D) for the osmotic solution loading/flushing. The channels were specifically sized for chosen commercially available fluidic connectors (QSM-M3-3-L, FESTO, Germany). The considered module also hosted a cylindrical slot (10 mm diameter, 1 mm height) to accommodate the bulging elastomer (see Figure 1E). This was glued to an annular surface (also visible in Figure 1B) that was adjacent to the cylindrical hole, thus allowing for bulge protrusion. The wall thickness for the annular surface was 0.5 mm (see Figure 1E), such that the bulge protrusions could be visually detected for bulge profiles higher than 0.5 mm. Moreover, potential leakage of the osmotic solutions toward

the RC was prevented by introducing an O-ring seal (DICHTA, Italy) with a 17 mm inner diameter and a 1 mm cross-section diameter. The O-ring slot is clearly visible in Figure 1B,D,E. In particular, sealing was achieved by fastening the OM sandwiching grids using 20 M1.4x2 precision screws (MISUMI Europe GmbH, Germany), whose slots and passages are visible in Figure 1A,B,C. A 3D view of the considered module featuring the fastened grids is shown in Figure 1F. Finally, a square flange with a side length of 40 mm (visible in Figure 1F) was chosen for the main module body. This was used for proper interfacing with the RC module. For ease of assembly, the flange was split into two parts, one of which was in the main body of the AC module.

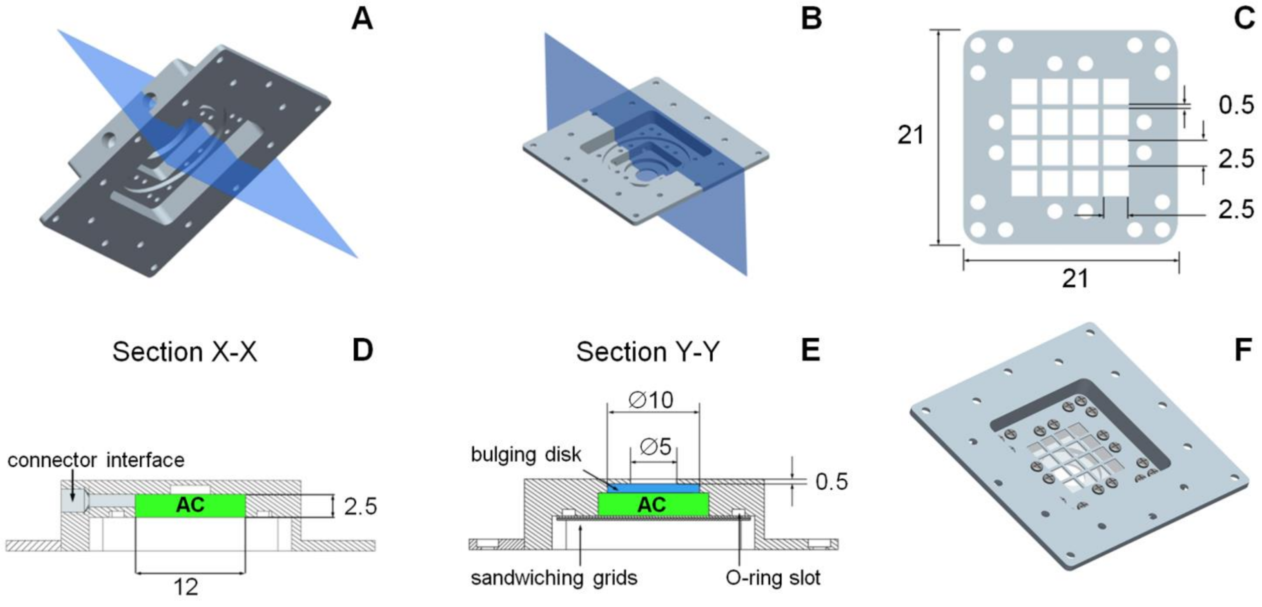

Figure 1. Design details of the actuation chamber module. (A,B) 3D views of the actuator module hosting, in particular, the actuation chamber. (C) Top view of an osmotic membrane sandwiching grid, also showing the passing-through holes for the fastening screws. (D) Section showing the interface for the loading/flushing connectors. (E,F) Section and 3D view of the main body of the module, also featuring the sandwiching grids. The bulging elastomeric disk is sketched in (E), for ease of illustration. Dimensions are shown in mm. (This figure replicates Figure 4 of the main text; it is reported here for ease of presentation.)

Reservoir chamber module design, and actuator assembly

The RC module consisted of a steel plate and a clear Plexiglas box (Figure 2). First, the 40x40x4.5 steel plate was designed to provide a suitable interface with the AC module. In particular, an O-ring seal (like that described above but with a 30 mm inner diameter) was introduced to prevent water leakage. Its slot is visible in Figure 2A. Sealing was achieved by fastening the RC and AC modules through 16 M1.4x3 precision screws (MISUMI Europe GmbH, Germany), whose slots and passages are visible (see Figure 1F and 2A). Furthermore, 8 M1.4x3 screw holes were added to the lateral surface of the plate to connect the actuator to its external supports. Finally, the plastic box was added as a simple cap allowing for visual inspection, especially of the OM, also through a proper slot in the steel plate (see Figure 2A). The box consisted of a base plate and four lateral ribs (8 mm width), one of which featured 2 passing through holes for water loading/flushing (see Figure 2B). An exploded view of the main components of the actuator is shown in Figure 2B.

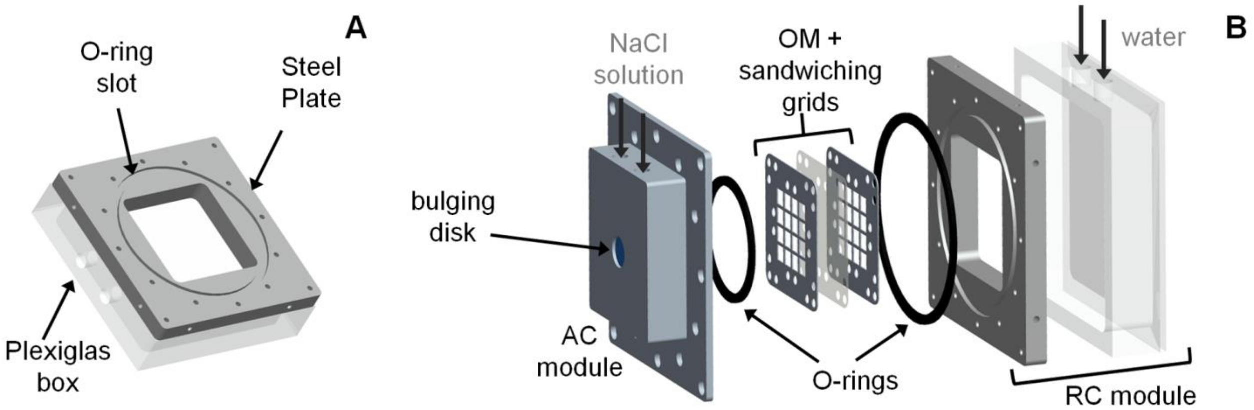

Figure 2. Design details of the reservoir chamber module, and actuator assembly. (A) 3D view of the reservoir chamber module. (B) Exploded view of the main components of the actuator. (This figure replicates Figure 5 of the main text; it is reported here for ease of presentation.)

Actuator fabrication

Laser cutting (CO_2 VERSAlaser VL S2.30 system, Laser&Sign Technology, Australia) was used to obtain the Plexiglas components of the RC module. These components were subsequently glued to one another and to the steel plate of the RC module using a bi-component epoxy glue. The same laser-cutting machine was exploited for profiling the OM to match the boundary shape of the corresponding sandwiching grids (including the 16 square slots and the 20 circles for the fastening screws), as well as for the elastomeric disk. The disk was subsequently glued to the main body of the AC module with a polychlorobutadiene glue. Furthermore, YAG laser cutting (SL 800 HS, LPKF Laser & Electronics, Germany) was used for the OM sandwiching grids. Finally, the AISI 316 stainless steel parts were fabricated using a CNC 5 axis micro-milling machine (HSPC, KERN, Germany).

References

1. Sinibaldi E, Puleo GL, Mattioli F, Mattoli V, Di Michele F, Beccai L, Tramacere F, Mancuso S, Mazzolai B (2013) Osmotic actuation modeling for innovative biorobotic solutions inspired by Plant Kingdom. Bioinspiration & Biomimetics, 8: 025002.
2. Mix AW, Giacomin AJ (2011) Standardized Polymer Durometry. Journal of Testing and Evaluation 39(4): 1-10.
